# High-precision cell-type mapping and annotation of single-cell spatial transcriptomics with STAMapper

**DOI:** 10.1186/s13059-025-03773-6

**Published:** 2025-10-07

**Authors:** Qunlun Shen, Kangning Dong, Shuqin Zhang, Shihua Zhang

**Affiliations:** 1 School of Mathematical Sciences, Fudan University, Shanghai, 200433 China; 2 School of Mathematics, Renmin University of China, Beijing, 100872 China; 3 State Key Laboratory of Mathematical Sciences, Academy of Mathematics and Systems Science, Chinese Academy of Sciences, Beijing, 100190 China; 4 Center for Applied Mathematics and Research Institute of Intelligent Complex Systems, Fudan University, Shanghai, 200433 China; 5 Shanghai Key Laboratory for Contemporary Applied Mathematics, Fudan University, Shanghai, 200433 China; 6 School of Mathematical Sciences, University of Chinese Academy of Sciences, Beijing, 100049 China; 7 Key Laboratory of Systems Health Science of Zhejiang Province, School of Life Science, Hangzhou Institute for Advanced Study, University of Chinese Academy of Sciences, Chinese Academy of Sciences, Hangzhou, 310024 China

## Abstract

In this paper, we develop a heterogeneous graph neural network, STAMapper, to transfer the cell-type labels from single-cell RNA-sequencing (scRNA-seq) data to single-cell spatial transcriptomics (scST) data. We collect 81 scST datasets consisting of 344 slices and 16 paired scRNA-seq datasets from eight technologies and five tissues to validate the efficiency of STAMapper. STAMapper achieves the best performance on 75 out of 81 datasets compared to competing methods in accuracy. STAMapper demonstrates enhanced performance over manual annotations, particularly at the boundaries of cell clusters, enables the unknown cell-type detection in scST data, and exhibits precise cell subtype annotations.

**Supplementary Information:**

The online version contains supplementary material available at 10.1186/s13059-025-03773-6.

## Background

Single-cell RNA sequencing (scRNA-seq) technologies allow us to study whole-transcriptome changes at the single-cell level to shape the diversity of cell types and their dynamic changes [1]. However, the spatial position information of single cells tends to be lost due to the dissociation during the sequencing process, which prevents us from understanding the relationship between gene expression and the tissue architecture and hinders us from deciphering the complex interactions between cells and their microenvironments under spatial context [2]. In recent years, spatial transcriptomics has rapidly evolved, enabling high-resolution gene expression mapping within tissue architecture. Alongside technological advances, a growing number of computational methods have been developed to analyze spatial transcriptomics data, focusing on various tasks such as spatial domain identification (e.g., IRIS [3], STAGATE [4]), detection of spatially variable genes (e.g., PROST [5], STANCE [6], STAMarker [7]), and spatial cell–cell communication (e.g., COMMOT [8], DeepTalk [9]). More recently, the emergence of single-cell spatial transcriptomics (scST) technologies such as MERFISH [10, 11], seqFISH [12], seqFISH + [13], osmFISH [14], STARmap [15], STARmap PLUS [16], NanoString [17], and Slide-tags [18], enables the profiling of gene expression with their spatial context at single-cell resolution.

The essential problem in scRNA-seq and scST data analysis is cell-type annotation (or cell typing) [19, 20]. The standard workflow [21] for cell-type annotation in scRNA-seq data is normalization, gene selection (usually top 2000 highly variable genes), dimensionality reduction, clustering, and assigning a cell type to each cluster according to the expression of known marker genes. When dealing with the scST data, the above workflow may fail since the sequencing quality of scST technologies is far lower than that of the mature scRNA-seq technologies. Specifically, these spatially resolved technologies typically focus on a pre-defined set of marker genes or genes relevant to biological processes (usually far fewer than 2000, Additional file1: Table S1). In the case of Slide-tags, a whole-transcriptome single-nucleus spatial technology, approximately 75% of nuclei are lost during sequencing [18]. These factors may lead to clustering instability and blurred cluster boundaries, resulting in inaccurate cell-type annotation. Moreover, as some of the markers for rare cell types may be absent in ST data, the annotation for the related cells could be challenged or overlooked. For the MERFISH hypothalamic data [22], annotation was performed by clustering all genes, refining neuronal subtypes through secondary clustering and doublet removal, and aligning clusters to scRNA-seq using correlation, classification, and anatomical constraints. This multi-step process is both time-consuming and complex. Therefore, accurate and fast annotation of single-cell ST data remains demanding and intricate.

With more scRNA-seq data available, reference-based scST annotation methods have been proposed to transfer cell-type labels to query datasets by leveraging the well-annotated reference dataset [23–25]. For example, scANVI employs a variational autoencoder architecture to learn a latent space of cellular states for both the scRNA-seq and scST data and utilizes the mean of the variational distribution associated with each cell to perform annotation [23]. RCTD utilizes a regression framework to model cell-type profiles in reference and account for platform effects, facilitating cell-type identification in spatial data [24]. Tangram maps scRNA-seq profiles onto ST data by maximizing the cosine similarity of the predicted and the observed expression matrix [25]. These methods can predict cell-type labels in datasets generated by MERFISH (scANVI) [26], Slide-seq (RCTD) [24], and STARmap (Tangram) [25]. However, these existing methods may fail to reveal fuzzy boundaries in scST annotations, and due to the lack of incorporating gene modeling, they cannot identify gene modules either shared by scRNA-seq and scST data or unique to each of them. Furthermore, to the best of our knowledge, there has not yet been a substantial number of prepared real datasets to evaluate the accuracy and robustness of the cell-type annotation methods across different ST technologies and tissue origins.

To this end, we develop STAMapper to accurately annotate cells from scST data by a heterogeneous graph neural network [27] with a graph attention classifier. Also, we collected 81 paired scRNA-seq and scST datasets with manual annotations and manually aligned them to evaluate the annotation accuracy. Extensive tests and comparisons with existing methods demonstrated the superiority of STAMapper in various biological applications, i.e., cell-type mapping of scST data, reannotation of blurred cell types, unknown cell-type detection, subtype annotation, and gene module extraction. Additionally, the collected data can serve as a benchmark for scST annotation, and the annotation results of STAMapper can serve as a reference.

## Results

### Overview of STAMapper

**Fig. 1:**
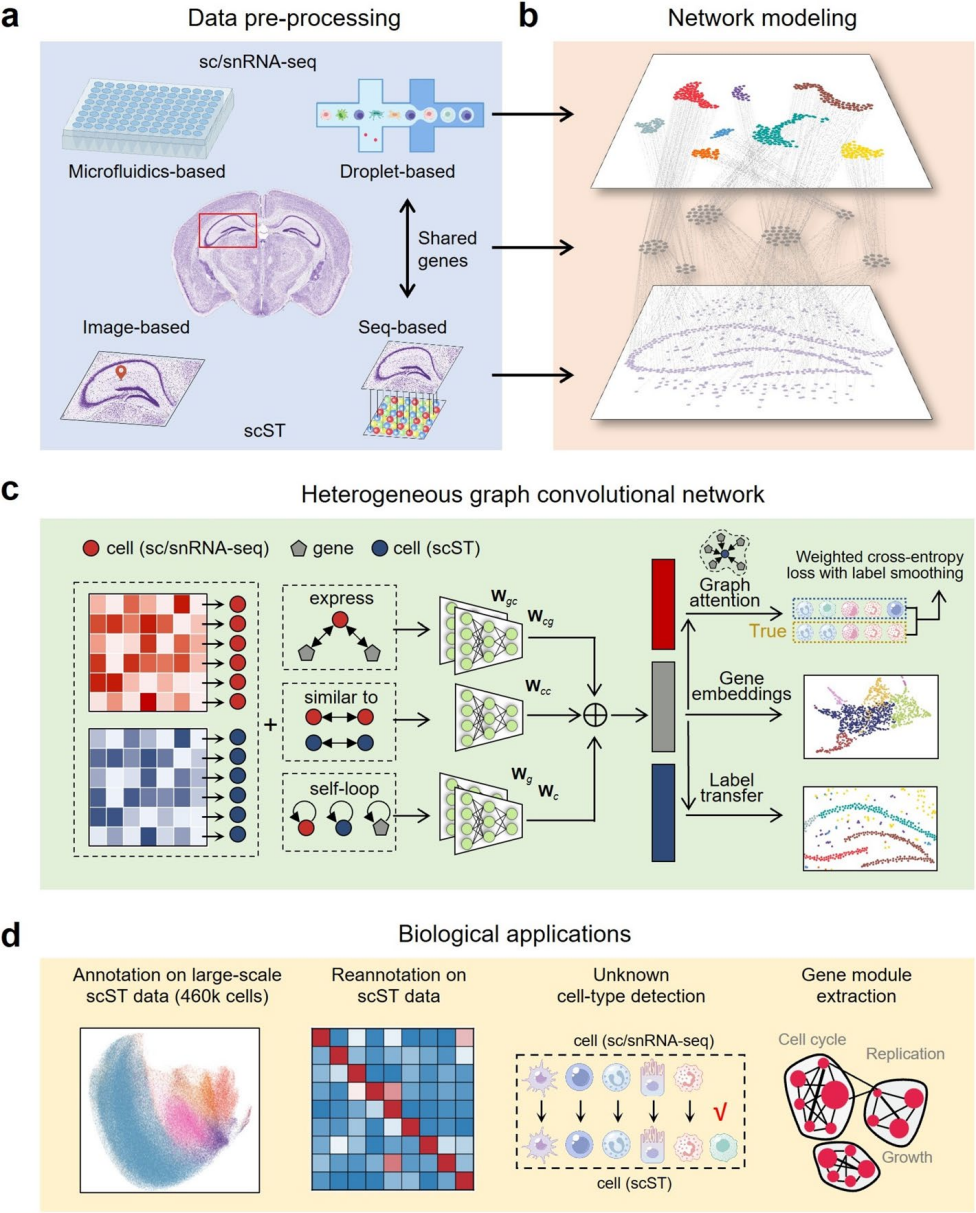
Illustration of STAMapper and its applications. **a** STAMapper can annotate scST data obtained from mainstream technologies such as image-based and seq-based by leveraging well-annotated sc/snRNA-seq data sequenced from microfluidics-based or droplet-based technologies. **b** STAMapper models genes and cells as two types of heterogeneous nodes and connects sc/scRNA-seq and scST data by their expression on the shared genes. **c** STAMapper takes the expression and the heterogeneous relationships of nodes as input. STAMapper then learns embeddings for cells and genes based on the information propagation mechanism on the heterogeneous graph network to fit cell labels from scRNA-seq data by using a graph attention classifier, ultimately utilizing the learned weights of information propagation on the graph to transfer cell labels on spatial data. **d** The output of STAMapper can be applied for annotation on large-scale scST data, reannotation on scST data, unknown cell-types detection, and gene module extraction

STAMapper takes a well-annotated scRNA-seq dataset and a scST dataset as input, where the two data matrices are normalized. STAMapper first constructs a heterogeneous graph, where the cells and genes are modeled as two distinct types of nodes and connected with edges based on whether the genes are expressed in the cells. Two cells from each dataset are connected if they exhibit similar gene expression patterns. Each node is connected to itself to indicate it utilizes the information from the previous step when updating its embedding (Fig. 1, Methods).

For each cell node, the initial input is the corresponding normalized gene expression vector. The gene nodes obtain their initial embedding by aggregating the input from the connected cell nodes (Methods). STAMapper updates the latent embedding of each cell or gene node based on the message-passing mechanism with information from its neighbors. It utilizes the embedding of gene nodes as the input of a graph attention classifier to estimate the probability of the cell-type identity, wherein each cell assigns varying attention weights to its connected genes. STAMapper uses a modified cross-entropy loss [28] (Methods) to quantify the discrepancy between the predicted and original cell-type labels for cells in the scRNA-seq dataset. Finally, through backpropagation, STAMapper updates the weights of parameters for different edges until the model converges. STAMapper determines gene modules based on the learned embeddings of gene nodes with the Leiden clustering algorithm [29] and applies the outputs of the graph attention classifier to assign cell-type labels to cells in the scST dataset.

### STAMapper enables accurate cell-type mapping for scST data

**Fig. 2:**
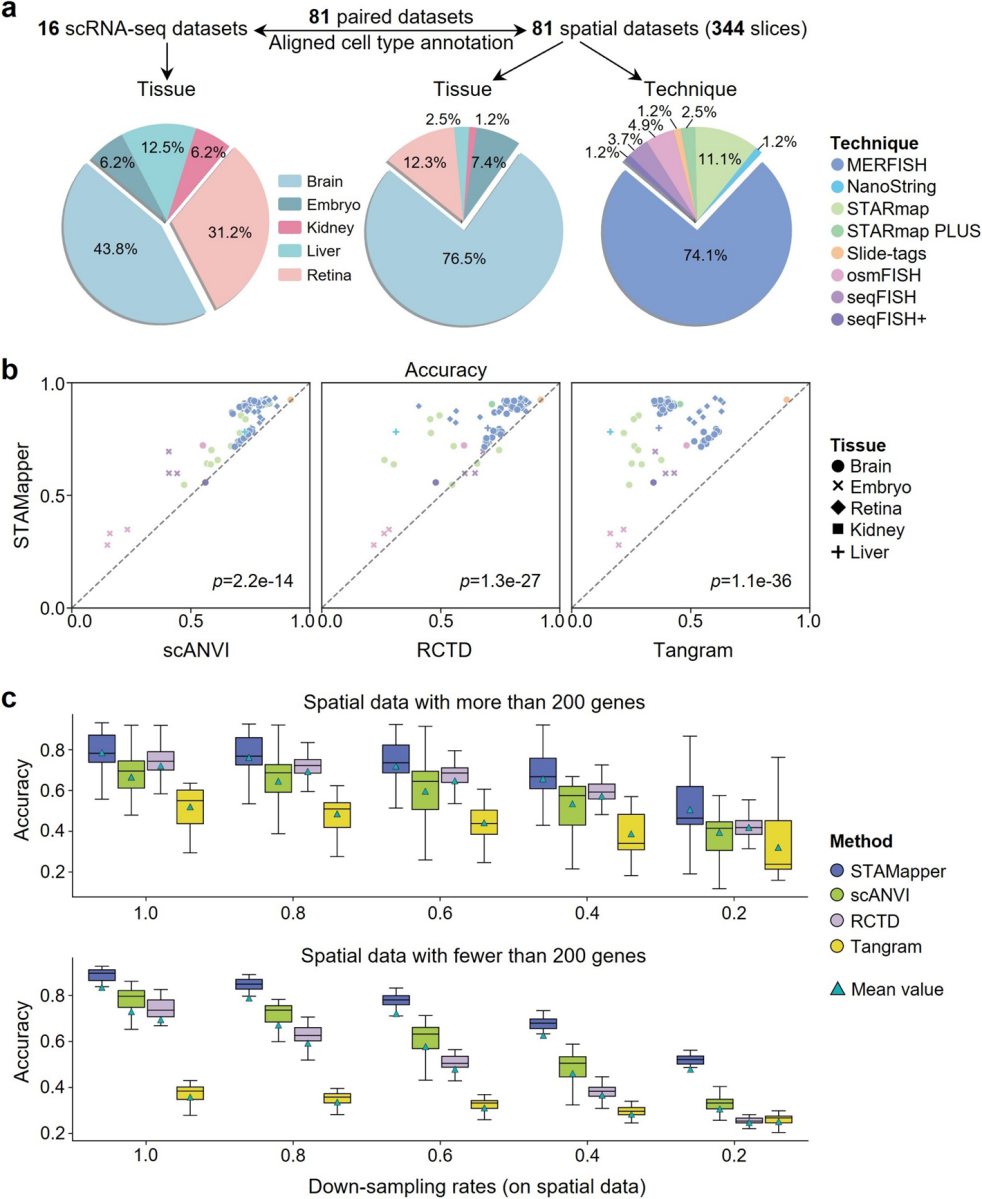
Benchmarking cell annotation performance of STAMapper. **a** Overview of all datasets used for evaluating the performance of STAMapper. We collected 81 single-cell spatial transcriptomics datasets comprising a total of 344 slices, where each dataset is matched with corresponding single-cell transcriptomics data (or scRNA-seq data). **b** Performance comparison of STAMapper and scANVI, RCTD, Tangram regarding cell annotation accuracy on 81 pairs of scRNA-seq and single-cell spatial transcriptomics datasets. *P* values were calculated by paired *t* test. **c** Performance comparison of the classification accuracies of STAMapper and three other methods on different down-sampling rates (1.0, 0.8, 0.6, 0.4, 0.2) for read counts, where the down-sampling rate of 1.0 means the raw data. The upper panel depicts spatial transcriptomics datasets with more than 200 genes for sequencing (47 datasets), while the lower panel corresponds to fewer than 200 genes (34 datasets)

We collected 81 single-cell ST datasets comprised of 344 slices and 16 paired scRNA-seq datasets from identical tissues. These scST datasets originate from eight single-cell ST technologies, i.e., MERFISH [10], NanoString [17], STARmap [30], STARmap Plus [16], Slide-tags [18], osmFISH [14], seqFISH [12], seqFISH + [13], and five different tissues, i.e., brain, embryo, retina, kidney, liver (Fig. 2a). All datasets come with manual annotations provided by the authors and the cell-type labels in paired scRNA-seq and spatial datasets aligned manually.

We quantitatively evaluated the cell-type annotation performance of STAMapper and the competing methods, i.e., scANVI [23], RCTD [24], and Tangram [25], in terms of accuracy, macro F1 score, and weighted F1 score (Methods). STAMapper demonstrated significantly higher accuracy in annotating cells from scST datasets compared with scANVI (*p* = 2.2e-14), RCTD (*p* = 1.3e-27), and Tangram (*p* = 1.3e-36) (Fig. 2b). STAMapper also achieved the best overall performance with the macro F1 score, compared with scANVI (*p* = 5.8e-16), RCTD (*p* = 7.8e-29), and Tangram (*p* = 1.5e-40) for the imbalanced cell-type distributions (Additional file 1: Fig. S1a). Also, STAMapper showed a significant advantage over all other methods in weighted F1 score (Additional file 1: Fig. S1b). scANVI ranked the second best across the three metrics. These results suggest that STAMapper exhibits the best annotation capability and proficiently identifies the rare cell types, which is crucial in cell-type annotation.

We evaluated the performance under poor sequencing quality with four different down-sampling rates on scST data. STAMapper consistently demonstrated the highest accuracy, macro F1 score, and weighted F1 score (Fig. 2c, Additional file 1: Fig. S1c, d). This trend is particularly distinct in scST datasets with fewer than 200 genes, where at a down-sampling rate of 0.2, STAMapper exhibited a much higher accuracy than the second-highest ranking method, scANVI (median 51.6% VS 34.4%) (Fig. 2c). For scST datasets with more than 200 genes, across all down-sampling rates (0.2, 0.4, 0.6, and 0.8), STAMapper still achieved the highest annotation accuracy, macro F1 score, and weighted F1 score, even though the performance margin was less superior (Fig. 2c, Additional file 1: Fig. S1c, d). RCTD demonstrated superiority in the raw data compared to scANVI (25 of 34 datasets) and comparable performance in the down-sampled data (Fig. 2c) for datasets containing more than 200 genes. scANVI tended to outperform RCTD on scST datasets with fewer than 200 genes in the raw data (41 of 47 datasets) and the down-sampled data regarding the accuracy and weighted F1 score. scANVI showed comparable performance with RCTD for datasets containing more than 200 genes, consistently outperforming RCTD on scST datasets with fewer than 200 genes in terms of macro F1 score (Additional file 1: Fig. S1c, d). Additionally, to mitigate the sensitivity of deep learning methods to hyperparameters, we tested the accuracy of scANVI under various hyperparameters (Additional file 1: Fig. S1e). We also compared STAMapper with CellTrek [31] and SeuratV4 [32], where STAMapper still achieved the best performance with significantly higher accuracy, macro F1, and weighted F1 scores (Additional file 1: Fig. S1f).

### STAMapper facilitates precise cell-type mapping within the retinal laminar structure

**Fig. 3:**
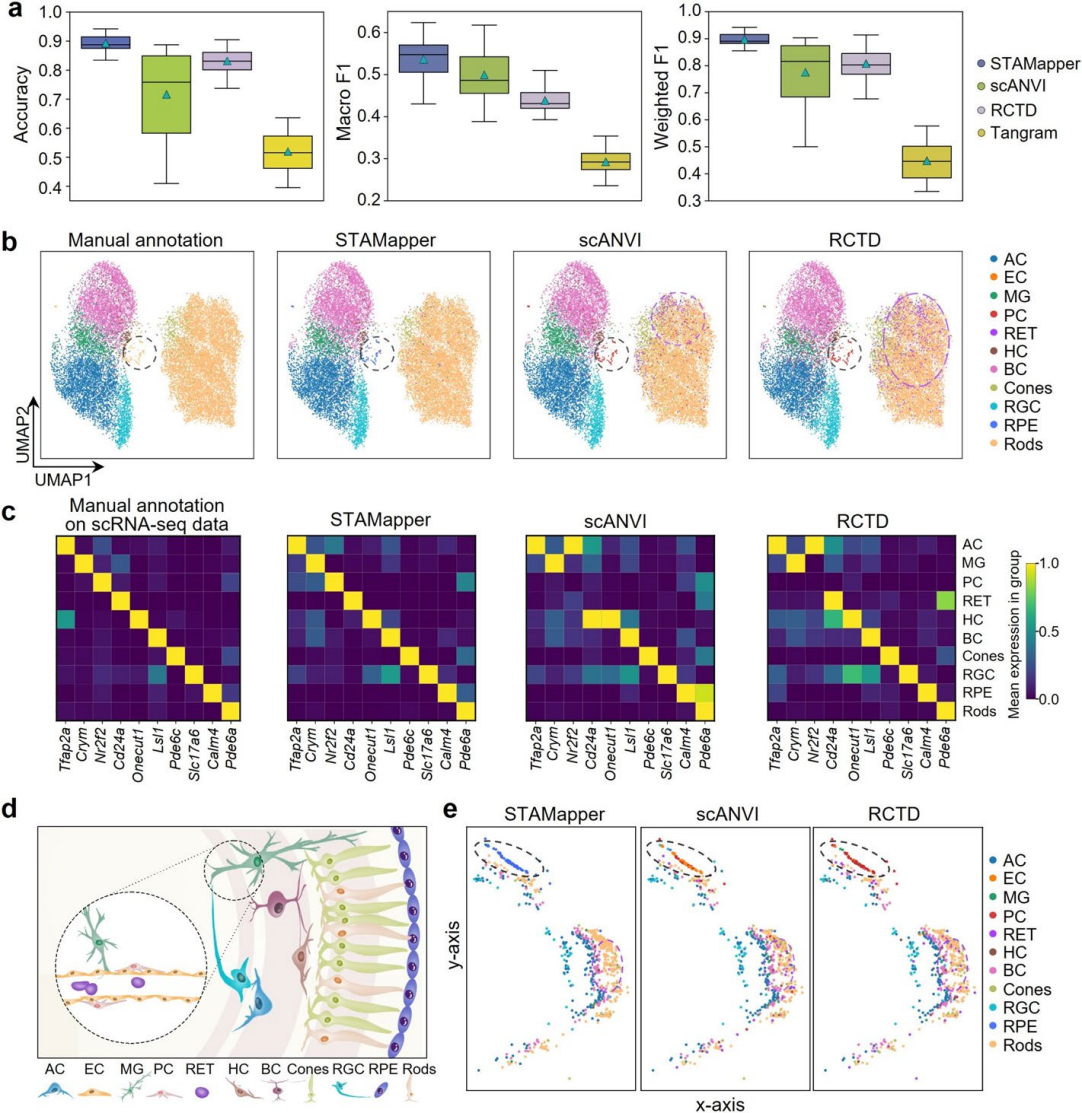
Application of STAMapper to MERFISH retina datasets. **a** Performance comparison of STAMapper and scANVI, RCTD, Tangram, where each box represents the method’s performance on the 50 paired datasets (five scRNA-seq datasets and ten single-cell spatial transcriptomics datasets).** b** UMAP plots of mouse retinal dataset (VZG105a_WT3) cells colored by the manual annotation and the prediction of STAMapper, scANVI, and RCTD using the mouse_LD_60 scRNA-seq dataset as the reference. *AC* amacrine cells, *EC* endothelial cells, *MG* Müller Glia, *PC* pericytes, *RET* reticulocyte, *HC* retinal horizontal cells, *BC* bipolar cells, *Cones* cone cells, *RGC* retinal ganglion cells, *RPE* retinal pigment epithelium, *Rods* Rod cells. **c** The heatmap of the marker expression for major cell types on the scRNA-seq dataset grouped by manual annotation and on the corresponding spatial transcriptomics dataset annotated by STAMapper, scANVI, and RCTD, respectively. **d** A schematic illustration of the distribution of cell types within the retina. **e.** Spatial organization of a slice from spatial transcriptomics dataset corresponding to (**b**), where cells are colored by the annotation by STAMapper, scANVI, and RCTD, respectively

We applied STAMapper to ten MERFISH datasets derived from the mouse retina [33], a highly organized tissue, on which we assessed the accuracy of annotations from the perspective of cell spatial positions beyond checking the expression of markers. Here, we selected five scRNA-seq datasets of the mouse retina collected from postnatal 0 h to 60 days (P60) as the reference data [34] to test the robustness of STAMapper. As expected, STAMapper consistently outperformed the other three methods on every spatial dataset according to accuracy, macro F1 score, and weighted F1 score (Fig. 3a, Additional file 1: Fig. S2a–c). Additionally, STAMapper exhibited the lowest variance in accuracy and weighted F1 score, demonstrating its robustness to changes in the reference data. Due to the poor performance of Tangram, we did not include it in subsequent analyses.

ScRNA-seq often allows for identifying some rare cell types since it can measure the expressions of most genes. However, some cell types identified in single-cell data, including endothelial cells (EC), pericytes (PC), retinal pigment epithelium (RPE), and reticulocyte (RET), do not appear in the manual annotations of spatial data, possibly due to the limited number of genes sequenced (Additional file1: Table S1) or inadequacies in clustering methods. In particular, we use WT3 sample as the considered spatial data, which only contained 368 genes, where not all the markers [34] of the cell types in scRNA-seq data were captured. In this case, STAMapper, scANVI, and RCTD were able to annotate some of these cell types. STAMapper annotated a group of rods (black dotted line in Fig. 3b), distant from the major cluster, as RPE but as PC by scANVI and RCTD. Additionally, scANVI and RCTD identified a cluster of RETs (purple dotted line in Fig. 3b and Additional file 1: Fig. S2d). To determine whether the annotated cell types indeed exist in the scST data, we first selected markers [34] in original spatial data and differential expressed genes for the newly annotated cell types and further validated by the scRNA-seq data (Fig. 3c). The expression of these genes confirmed the accurate annotation of STAMapper. The PCs annotated by scANVI and RCTD did not express *Nr2f2*, and the RETs annotated by scANVI also demonstrated inconsistency in the expression of *Cd24a,* which were two genes showing highly differential expression in the corresponding cell types from scRNA-seq data (Fig. 3c). This indicated that there could be inaccuracies in their annotation.

We further examined their spatial locations to validate the annotation of newly discovered cell types. Here, scANVI and RCTD identified the cells in the outermost layer of the retina as ECs and PCs (black dashed ellipse in Fig. 3e), which does not align well with their real anatomical location [35, 36] (the inner layer of retina) (Fig. 3d and Additional file 1: Fig. S2f–g), while STAMapper annotated this group of cells as RPE cells, which expressed the corresponding markers in the appropriate anatomical positions [37] (Fig. 3c–e and Additional file 1: Fig. S2e). Different from STAMapper, scANVI and RCTD mistakenly annotated PCs among the rods (black dashed ellipse in Fig. 3e), which should be located in the retina’s inner side, consistent with our observations on Uniform Manifold Approximation and Projection (UMAP) (Fig. 3b and Additional file 1: Fig. S2f). In addition, scANVI and RCTD wrongly identified many RET cells between rods (purple dashed ellipse in Fig. 3e), which should be located at the most inner part of the retina (Fig. 3d and Additional file 1: Fig. S2d). Similar situations can be observed across the other six slices (Additional file 1: Fig. S2h–m). Overall, STAMapper uncovered the cell types that clustering alone fails to identify in spatial retinal data by making full use of the detailed annotations from scRNA-seq data. More importantly, the annotation of STAMapper perfectly aligned with the retinal architecture, spanning from the outer retinal cells to the inner supporting cells.

### STAMapper corrects the blurred cell-type annotations caused by clustering-induced boundaries

**Fig. 4:**
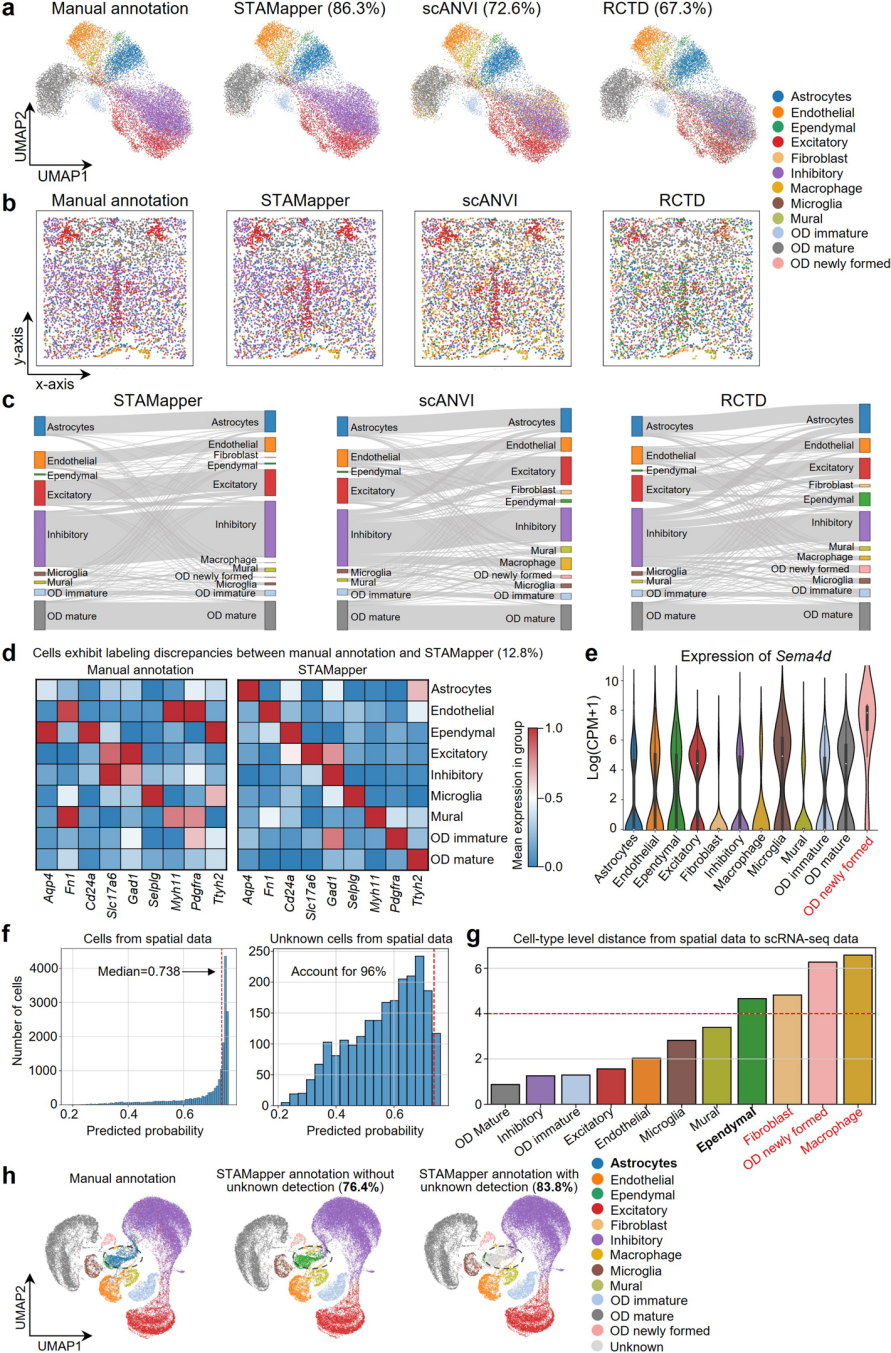
Application of STAMapper to MERFISH hypothalamic dataset. **a** UMAP plots of mouse hypothalamic dataset colored by the manual annotation and the prediction of STAMapper, scANVI, and RCTD, respectively. **b** Spatial organization of a slice from mouse hypothalamic dataset corresponding to (**a**), cells are colored by the manual annotation and the prediction of STAMapper, scANVI, and RCTD, respectively. **c** Sankey plot showing the accuracy of the cell-type annotation by STAMapper, scANVI, and RCTD, respectively. The left side of the Sankey plot represents manual annotations, while the right side shows the predicted results. The height of each linkage line reflects the number of cells. **d** Heatmap plot of marker expression for major cell types presented in manual annotation with mismatched labels between manual annotation and STAMapper. **e** Expression levels of *Sema4d* (a marker of OD Newly formed**)** across different cell types (annotated by STAMapper). **f** The predicted probability of STAMapper for cells from spatial data (left panel) and unknown cells from spatial data (right panel), the red dash line indicates x = 0.738 in both panels. **g** Cell-type level distance from spatial data to single-cell data on cell embeddings learned by STAMapper. Bold indicates unknown cells were predicted as this specific cell type, and red denotes cell types present in single-cell data but not annotated in spatial data by manual annotation. **h** UMAP plots of the co-embedding of scRNA-seq and spatial data learned by STAMapper. Cells are colored by manual annotation, STAMapper prediction without unknown detection, and STAMapper prediction with unknown detection. The percentages in parentheses represent the predicted accuracy

We further applied STAMapper to the MERFISH hypothalamic data (ID = 15) collected from mouse brain [22] with the reference scRNA-seq data from the same study to test whether it can help correct the cell-type annotations at the boundaries detected by clustering algorithms. STAMapper achieved the highest annotation accuracy (86.3%) compared with scANVI (72.6%) and RCTD (67.3%). Specifically, scANVI mistakenly identified many macrophages mixed with inhibitory neurons and some fibroblasts at the boundary of OD mature cells. RCTD mistakenly annotated some ependymal cells mixed with inhibitory neurons and excitatory cells. In contrast, the annotation of STAMapper avoided the mixing of cell types between two non-transitional cells and showed smoothness at the boundaries of the clusters (Fig. 4a and Additional file 1: Fig. S3a). Taking a close look at a slice of this sample, we discovered that STAMapper accurately recovered the “arrow-like” excitatory neurons in the middle of the slice, whereas RCTD mistakenly annotated them as a mixture of excitatory neurons and ependymal cells. STAMapper correctly identified the cellular microenvironment dominated by inhibitory neurons, and scANVI and RCTD failed (Fig. 4b and Additional file 1: Fig. S3a). For the remaining three slices, STAMapper maintained the highest degree of consistency with manual annotations (Additional file 1: Fig. S3b–d). The Sankey plot of the entire sample demonstrated that scANVI and RCTD incorrectly predicted many inhibitory neurons to other cells like astrocytes, macrophages, and excitatory neurons (Fig. 4c). scANVI predicted a considerable number of macrophages annotated as inhibitory neurons manually. However, these cells did express the inhibitory neuron marker *Gad1*, indicating the annotation errors by scANVI (Additional file 1: Fig. S3e).

Despite STAMapper exhibited the highest annotation accuracy, some cells showed inconsistencies with manual annotations. These cells tended to be located at the boundaries of cell clusters, e.g., around microglia (Fig. 4a). We examined the cell types already presented in the manual annotations. Some cell types did not express the corresponding markers from the original study [22]. Yet the annotation of STAMapper aligned precisely with the expression of marker genes (Fig. 4d). Also, STAMapper identified OD newly formed cells, which were not previously recognized in manual annotations, verified by highly expressing their marker gene *Sema4d* [38] (Fig. 4e).

We further extended STAMapper to detect unknown cell types in spatial data. Here, we removed astrocytes from the scRNA-seq dataset and assumed to identify them as unknown cells in the spatial data. We defined the potentially unknown cells by the following two rules: (i) predicted probability less than the median value of all cells from spatial data (global prediction confidence), and (ii) the cell-type level distance from scST data to scRNA-seq data higher than a user-defined threshold (local distributional discrepancy) (Fig. 4f, g, Methods). In addition to tuning the threshold, we offered the option to specify low-confidence cell types, which offers flexibility and can be tuned based on dataset characteristics, e.g., by inspecting the expression of marker genes in cell types that display a large distance. In this scenario, STAMapper without unknown cell detection primally achieved an accuracy of 76.4%, where it annotated astrocytes as ependymal reasonably, which is another type of glial cell. Leveraging the mechanism of unknown detection, STAMapper successfully annotated astrocytes as unknown cells and increased the accuracy to 83.8% (Fig. 4h). We also performed another test, where we removed OD immature cells from the scRNA-seq dataset, and STAMapper continued to annotate these cells as unknown correctly (Additional file 1: Fig. S3f–h). We also removed endothelial cells (Additional file 1: Fig. S3i–k), inhibitory and excitatory neurons (Additional file 1: Fig. S3l–n), OD mature and astrocytes (Additional file 1: Fig. S3o-q) from the scRNA-seq dataset, and STAMapper continued to annotate these cells as unknown and largely improved the accuracy of annotation. Therefore, STAMapper could rectify cell types at classification boundaries that are challenging to identify with clustering algorithms and detect unknown cell types in spatial data.

### STAMapper achieves precise annotations aiding in deciphering the tumor microenvironment

**Fig. 5:**
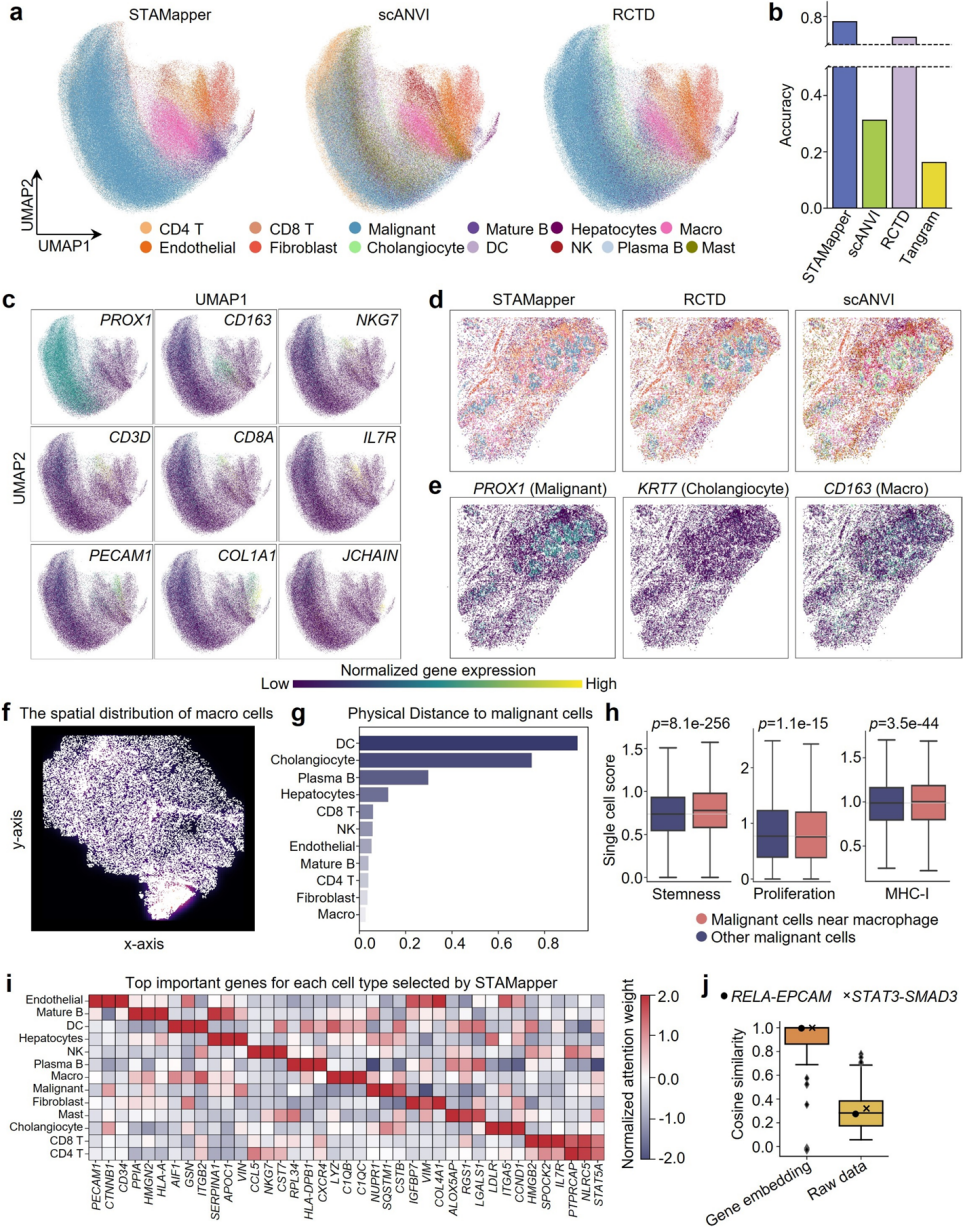
Application of STAMapper to Nanostring HCC dataset. **a** UMAP plots of the human HCC dataset colored by STAMapper, scANVI, and RCTD, respectively. *Macro* macrophage, *NK* natural killer, *DC* dendritic cell. **b** Accuracy of STAMapper, scANVI, RCTD, and Tangram on human HCC dataset. **c** UMAP plot of the normalized marker expression corresponding to major cell types. **d** Spatial organization of ROI 1, cells are colored by the annotation of STAMapper, RCTD, and scANVI, respectively. **e** The normalized marker expression on ROI 1. **f** Density plot for the distribution of macro cells, annotated by STAMapper. **g** Physical distance of immune cells to malignant cells, with cells being annotated by STAMapper. **h** Boxplot for the scores of selected pathways (stemness, proliferation, and MHC-I) on malignant cells near macro and other malignant cells. **i** Heatmap displaying genes with the highest normalized attention weights, categorized by each cell type. We aggregate the normalized attention weights from that gene to all cells (Methods) belonging to the cell type and compute their average as the cell type’s normalized attention weights. These scores reflect the gene’s overall contribution to the annotation of that cell type. **j** Cosine similarity of the gene embedding pairs learned by STAMapper, where gene pairs were TFs collected from hTFtarget. *RELA*-*EPCAM* and *STAT3*-*SMAD3* were validated to exist in HCC malignant cells in the literature

We applied STAMapper to the human hepatocellular carcinoma spatial data sequenced by NanoString technology [17], and the scRNA-seq data originated from the same type of tumor lesions as a reference [39]. Compared to scANVI and RCTD, the annotation of STAMapper could reveal distinct boundaries between different cell types and achieve the highest consistency with the annotations provided by the authors (Fig. 5a, b). The expression of markers aligned closely with the corresponding major cell types [40], i.e., *PROX1* (Malignant), *CD163* (Macro), *NKG7* (NK), *CD3D* (T cell), *CD8A* (CD8 T), *IL7R* (CD4 T), *PECAM1* (Endothelial), *COL1A1* (Fibroblast), *JCHAIN* (Mature B) (Fig. 5a, c). We selected two regions of interest (ROIs) in the tissue section (Additional file 1: Fig. S4a). ROI 1 was mainly malignant cells marked with highly expressed *PROX1*. scANVI annotated more DCs in this region, while RCTD annotated some cholangiocytes. However, the markers corresponding to these two cell types are barely expressed in this region (Additional file 1: Fig. S4b, c). ROI 2 appeared as a region mixed with immune cells and malignant cells, where numerous cells are distributed in a circular formation in the middle of this region (Fig. 5d). STAMapper annotated it as a microenvironment where macrophages enveloped malignant cells, T cells located in the outer side of this structure, and the exterior layer consisted of mature B cells. RCTD annotated the innermost layer with cholangiocytes surrounded by malignant cells, whereas scANVI annotated the innermost layer as mainly cholangiocytes with very few malignant cells. The expression of *PROX1* validated the presence of malignant cells in the innermost layer, and its expression is consistent with the annotations of STAMapper (Fig. 5d, e). Meanwhile, cells in this region barely expressed *KRT7*, a marker of cholangiocyte [41], indicating that the annotations by RCTD and scANVI were incorrect (Fig. 5e). Interestingly, all three methods agreed that there exists a reticular structure of macrophages in this area (Additional file 1: Fig. S4d), and in fact, they surrounded the malignant cells, forming a unique microenvironment (Fig. 5d).

We found that macrophages were widely present in the sections, especially at the edges of the malignant cells, and they were the immune cells closest to the malignant cells (Fig. 5f, g). Macrophages are considered to play a crucial role in tumor immune evasion and also serve as pro-inflammatory mediators [42]. To investigate the role of this unique malignant-macrophage microenvironment, we classified the malignant cells into two groups, i.e., malignant cells near macrophages and others. The malignant cells near macrophages showed higher stemness [43] and MHC-I score [44] but lower proliferation [40], indicating that, for malignant cells near macrophages, the increase in stemness was not due to proliferation, or they were less likely to be recognized by T cells. Therefore, it should be due to the influence of macrophages (Fig. 5h).

STAMapper could identify the most critical genes for each cell type and most of them are marker genes or differentially expressed genes, e.g., *PECAM1* for Endothelial [45], *HLA-A* for Mature B [46], *NKG7* for NK [47], *C1QB* for Macro [48], etc., which enhanced the model’s interpretability (Fig. 5i, Methods). Additionally, STAMapper provided gene embeddings that enhanced the similarity of functionally related gene pairs collected from hTFtarget [49]. We found that two TF-target pairs, validated in the literature [50, 51], exhibited cosine similarities close to 1 (Fig. 5j, Methods). Collectively, STAMapper could provide precise annotations with interpretability, helping to discover and explain the complex tumor microenvironment.

### STAMapper reveals the cellular subpopulation localization within the layered structure of the human cortex

**Fig. 6:**
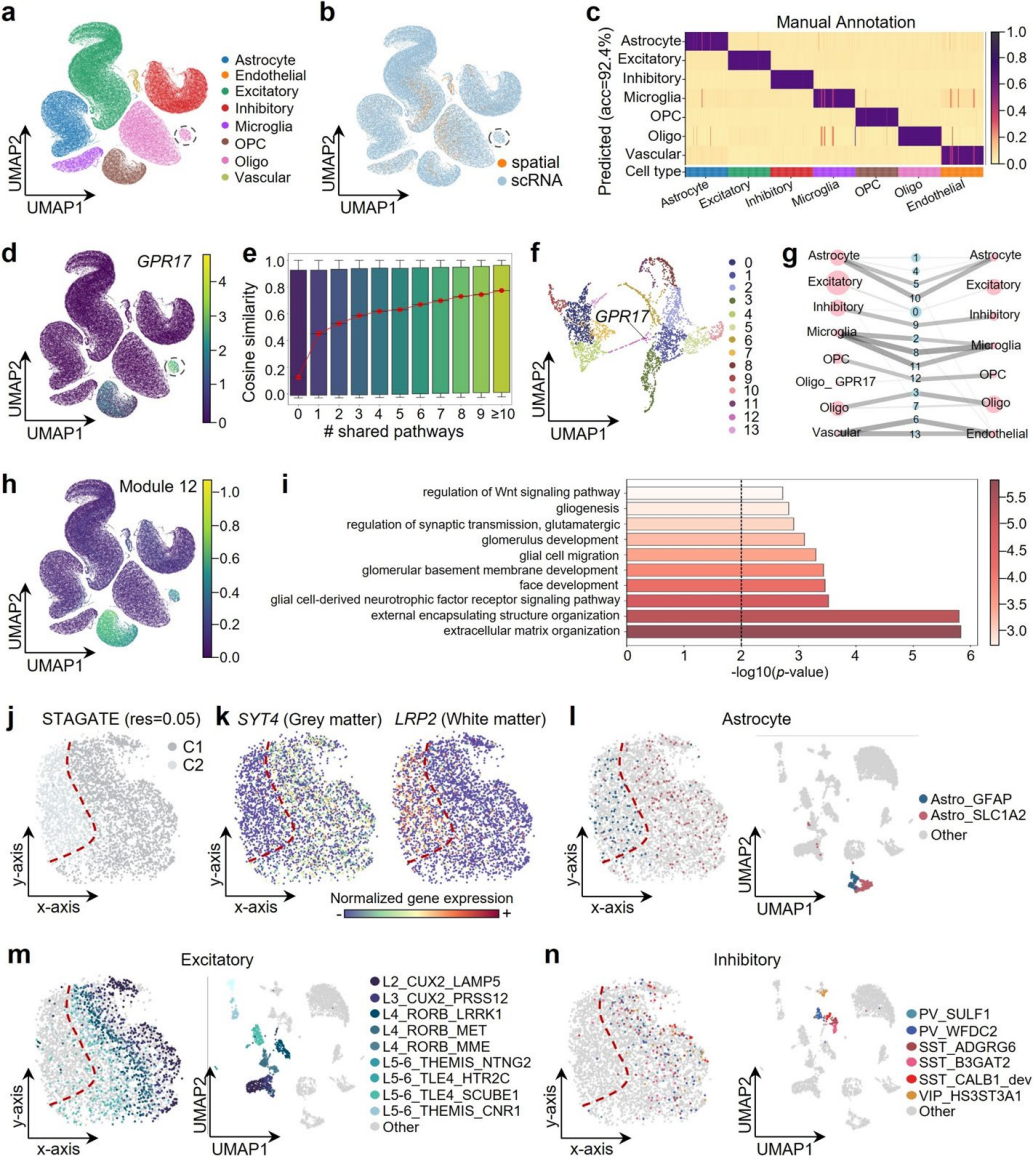
Application of STAMapper to Slide-tags human prefrontal cortex dataset. **a**, **b** UMAP plot for the co-embedding of scRNA-seq and spatial dataset learned by STAMapper. Cells are colored based on the prediction of STAMapper (**a**) and the origin of the datasets (**b**), respectively. *Oligo* oligodendrocytes, *OPC* oligodendrocyte progenitor cells. **c** The predicted cell-type probabilities for each cell (each column) in the spatial data. A maximum of 50 cells was subsampled from each type for visualization. **d** UMAP plots showing the co-embedding of the scRNA-seq and spatial dataset learned by STAMapper, cells are colored by the normalized expression levels of *GPR17*. **e** Boxplots of the cosine similarity between gene embedding pairs grouped by the number of shared pathways. **f** UMAP plot for the distribution of gene embedding. Genes are colored by clusters identified through the Leiden algorithm. **g** Abstracted graph of the heterogenous cell-gene graph, where nodes represent cell types (pink) or gene modules (blue). Node size reflects the number of cells in a cell type or genes in a module. Edge width varies with the average expression levels of cell types linked to gene modules, determined by STAMapper. **h** UMAP plots showing the co-embedding of scRNA-seq and spatial dataset learned by STAMapper, cells are colored by the normalized expression levels of Module 12. **i** Enrichment analysis of gene module 12 related to Oligo_GPR17 cells. **j** Spatial organization of cells from the spatial dataset. Cells are clustered by STAGATE with resolution = 0.05. **k** The Normalized expression of *SYT4* (marker gene of grey matter) and *LRP2* (marker gene of white matter). **l**–**n** Spatial organization and UMAP plot of astrocyte (**l**), excitatory (**m**), and inhibitory (**n**) Subtypes predicted by STAMapper from the spatial dataset, Subtypes with more than 20 cells are shown. The UMAP coordinates are calculated from the expression of the spatial data

We applied STAMapper to the human prefrontal cortex (PFC) sequenced by Slide-tags, a whole-transcriptome single-nucleus spatial technology [18], and the single-nucleus RNA dataset profiled for PFC samples as a reference [52]. STAMapper successfully aligned identical cell types across two datasets, including glial cells and neurons (Fig. 6a, b), and provided precise annotations for the scST data (Fig. 6c). The vascular cells (VCs, from the scRNA-seq data) and endothelial cells (ECs, from the scST data) were aligned well. That is reasonable since EC is a subtype of VC, and they both expressed *ITIH5*, a marker of ECs [18] (Additional file 1: Fig. S5a). Utilizing the cell embeddings provided by STAMapper, we discovered a distinctive subset of oligodendrocytes (oligo) in the single-cell data that expressed *GPR17* (Fig. 6b, d). This gene acts as an intrinsic timer of oligodendrocyte differentiation and myelination [53], suggesting these cells may be in a state of differentiation or development (labeled as Oligo_GPR17 for further analysis).

To further understand whether the gene embeddings provided by STAMapper captured biological meanings for each pair of input genes, we explored relationships between the number of shared pathways (Reactome [54] and cell type signatures from MSigDB [55]) and the gene cosine similarities (Methods). The cosine similarities were relatively low (median = 0.12) for gene pairs not presented in any biological pathway. We observed a clear gap in the cosine similarities for gene pairs that shared a pathway (median = 0.45). The more pathways shared, the higher their cosine similarities were (Fig. 6e). The cosine similarity achieved a median value of 0.77 for gene pairs occurring in at least ten pathways. Such a trend also existed in the raw data and was enhanced by STAMapper (Additional file 1: Fig. S5b). We next extracted 14 gene modules from these informative gene embeddings (Fig. 6f, Methods). They shared similar transcriptional patterns in the common cell types identified in both scRNA-seq and spatial data (Fig. 6g). Module 12 tended to be expressed in the oligodendrocyte progenitor cells (OPCs) and Oligo_GPR17 but not in oligos, and was enriched in pathways related to differentiation and development, i.e., glial cell-derived neurotrophic factor receptor signaling pathway, glial cell migration, gliogenesis and regulation of Wnt signaling pathway [56] (Fig. 6g–i, Methods). These results further indicated that Oligo_GRP17 represents a cell type distinct from traditional oligos, potentially arising from the differentiation of OPCs.

The layered structure is a main characteristic of the cortex [57]. We next performed subtype annotations on the scST data to explore their association with the cortex structure (Additional file 1: Fig. S5c, f). To reveal the hierarchical structure of the cortex, we initially employed the clustering method STAGATE [58] at a low resolution. STAGATE divided the section into white matter (WM) and gray matter (GM), verified by corresponding markers [59] (Fig. 6j, k, Methods). The astrocytes expressing *GFAP* were associated with WM in mice [60], and *SLC1A2* tended to express in the GM area [61]. STAMapper correctly annotated Astro_GFAP and Astro_SLC1A2 to the corresponding regions (Fig. 6l). The Oligos prefer to locate at WM [62] (Additional file 1: Fig. S5d). On the contrary, the excitatory neurons and inhibitory located at GM are consistent with a recent study [63] (Fig. 6m, n). Additionally, the subtypes of excitatory neurons exhibited layer-specific localization from the GM border to the GM/WM junction (from L2 to L6), consistent with the result of STAGATE (resolution = 0.3) (Fig. 6m, Additional file 1: Fig. S5e). These results illustrated that STAMapper provided gene embeddings with biological meanings, facilitating the identification of shared or unique gene modules across datasets. Also, STAMapper accurately annotated structurally related cell subtypes, aiding in understanding their positional context within tissues.

## Discussion

Precise annotation of single cells from the scST data is essential for understanding the complex interactions between cellular functions and their physical locations within tissues and organs. Here, we developed an accurate and user-friendly cell-type annotation method, STAMapper, which can be seamlessly integrated with the standard workflow of the package Scanpy [64].

The primary reason for the success of STAMapper lies in its utilization of both reference and query cells within a unified heterogeneous graph, where all cells share the same node type and connect to a common set of gene nodes. These gene nodes act as shared anchors that bridge reference and query data, enabling the model to capture consistent expression patterns across datasets. This encourages the alignment of both reference and query cells in a shared latent space. Moreover, STAMapper learns the edge weights that encode the relationship of cell embedding and cell types by training on scRNA-seq data, and these weights are shared with the scST dataset. Consequently, once the scRNA-seq data has established a robust mapping between expression profiles and cell types, this mapping can be directly leveraged to generate accurate annotations for the scST data*.* Also, it adopts a graph attention classifier to ensure that each cell pays more attention to genes that are more biologically related during the classification. However, STAMapper does not incorporate the spatial information of the cells in the current version. Although it is straightforward to model the spatial information within STAMapper by connecting new edges for spatially adjacent cells, we found that this led to an accuracy loss of about 0.4% with statistical significance (Additional file 1: Fig. S6a). This could be because many spatially adjacent cells are not of the same cell type. Especially, in median, only about 2 out of its 5 spatial neighbors belong to the same cell type for all datasets. As a result, modeling spatial adjacency alone, without considering transcriptomic similarity, can introduce noise and negatively impact model accuracy. Therefore, how to reasonably utilize spatial information to enhance the accuracy of annotations remains a challenging problem.

Benchmarking is quite an important issue when discussing the accuracy and robustness of a method. However, different computational methods utilize different datasets, which complicates comparing them fairly. In this study, we gathered 81 scST datasets from five distinct tissues and eight different technologies with corresponding scRNA-seq datasets as references. We carefully examined each dataset using manual validation based on canonical marker genes. These standardized datasets are promised to become a benchmark process for testing methods that are proposed to annotate scST-seq data. Additionally, STAMapper achieved superior performance to competing methods, demonstrating its superior efficacy. We also evaluated the performance of different annotation methods on a colon adenocarcinoma (COAD) dataset profiled by Xenium, a popular subcellular platform [65]. STAMapper still achieved the highest accuracy (93.91%) compared with scANVI (66.33%), RCTD (92.23%), and Tangram (37.89%) (Additional file 1: Fig. S6h).

In this study, we concentrated on cell-type annotation for scST datasets. Given a well-annotated scRNA-seq dataset as a reference, STAMapper can accurately annotate cell types in scST data and is robust to different spatial sequencing technologies and diverse tissues. Furthermore, while some deep learning methods may be sensitive to hyperparameter settings, tests on variations in hidden units and hidden layers revealed that STAMapper is robust to such changes (Additional file 1: Fig. S6b), further highlighting its potential for broad applicability. We also conducted additional ablations of heterogeneous edge types or processes that can be removed from STAMapper. Specifically, we (i) removed the “cell_similar_to_cell” edge type, (ii) disabled the filter on the “cell_similar_to_cell” edges that connect different cell types, and (iii) removed the gene_self_loop edges. These modifications lead to a 0.7–3.0% drop in annotation accuracy (Additional file 1: Fig. S6c), demonstrating that all of these edges and steps are essential. For STAMapper, the runtime increases linearly with the number of cells. When processing the largest dataset (460 K cells), STAMapper completed the task in about 5.3 h, using only 11.29 GB of GPU memory and 38.99 GB of system memory. This demonstrates that STAMapper can be efficiently applied to large-scale datasets (Additional file 1: Fig. S6d). On average, STAMapper demonstrated the fastest runtime, scANVI was slightly slower, and RCTD was much slower (Additional file 1: Fig. S6e). In terms of memory consumption, RCTD used the least memory, followed closely by STAMapper, but scANVI consumed substantially more memory than the other two methods (Additional file 2:Table S3). Overall, STAMapper offers both fast runtime and relatively low memory usage, indicating strong potential for scaling to large datasets. Further, to assess how cell numbers affect the analysis, we defined a metric, cell ratio between scRNA-seq reference and spatial data (CRSS), as the number of cells in the reference dataset divided by the number of cells in the spatial dataset. We observed a negative trend between CRSS and STAMapper accuracy; however, it did not reach statistical significance (Additional file 1: Fig. S6f). One potential limitation of STAMapper is that it utilizes only one scRNA-seq dataset as a reference, which could lead to the omission of certain cell types if the sequenced cells in the reference are not comprehensive. Future work may consider using multiple references to annotate spatial data to improve the annotation.

Selecting spatially variable genes has become a popular topic in recent years. However, the existing methods, e.g., SPARK-X [66], STAMarker [7], and spatialDE [67], are designed for spatial transcriptomics technologies with lower resolution, i.e.,10 × spatial transcriptomics [68]. In scST data, cell type can be employed to identify genes exhibiting spatial variation within the same cell population. STAMapper could help with an attention score to reflect the importance of each gene to a cell. If such a score undergoes a drastic change along spatial positions within a specific cell type, it could be potentially identified as a spatially variable gene relating to this cell type. Also, STAMapper does not include spatial information. A recent proposed method, CAESAR [69], integrates histology images and spatial location information into a low-dimensional space to characterize the gene-cell relationship (Additional file 1:Supplementary Note 1). We expect STAMapper to be extended in this direction.

## Methods

### Data description

We collected 81 scST datasets sequenced by different technologies, including MERFISH, seqFISH, seqFISH +, osmFISH, STARmap, STARmap PLUS, NanoString, and Slide-tags (Fig. 1, Additional file1: Table S1). For the NanoString HCC data, the authors provided the annotation by an unpublished method InSituType, and we used this annotation as ground truth. For the other 80 scST datasets and all the scRNA-seq datasets, the authors provided manual annotations that served as ground truth. We also collected 16 scRNA-seq datasets sequenced by different technologies, including 10 × Chromium, Droplet-microfluidic and STRT/C1 (Fig. 1, Additional file1: Table S1). The cell types across scRNA-seq and scST datasets were unified manually through corresponding literature and Cell Ontology [70]. The unification process between the scRNA-seq and scST datasets can be found in section “Data availability” below.

### Detection of differentially expressed genes

Differentially expressed genes (DEGs) were identified based on *t* test, ranked based on the adjusted *p* value, and filtered with a threshold of < 0.05.

### Data preprocessing

In all datasets, we first normalized the library size for each cell and then logarithmized the expression data with a pseudo-count. For scRNA-seq datasets, we then selected the top 2000 highly variable genes (HVGs) and calculated the top 50 differentially expressed genes (DEGs) for each cell type as input. For the scST datasets with more than 2000 genes, we used the same strategy for selecting HVGs. For the selection of DEGs, we first pre-clustered the spatial data by the Leiden algorithm [29] (resolution = 0.4) and then calculated the top 50 DEGs for each cell cluster. For the scST datasets with fewer than 2000 genes, we used the expression of all the genes as input. Finally, we scaled each gene to unit variance and zero mean value. All the preprocessing steps were implemented using the built-in functions in the package Scanpy [64].

### Construction of the heterogeneous graph

STAMapper uses a heterogeneous graph to model the two datasets with genes and cells as two types of nodes (Fig. 1a). We use a combination of HVGs and DEGs to determine the set of gene nodes, aiming to balance the representation of overall expression variability of genes that are highly informative for distinguishing cell type. Specifically, we used the intersection of selected genes (DEGs and HVGs) between scRNA-seq and scST datasets as the gene nodes. We have five types of heterogeneous edges. Specifically, for each cell node and gene node, we have the edge connected to itself named “cell_self_loop” and “gene_self_loop.” They help utilize information from the previous step in the training process. We also have “cell_similar_to_cell” edges connected to similarly expressed cells with the *k* nearest neighbor strategy (based on their expression vector, *k* = 5 by default) within each dataset. For a scRNA-seq dataset, we filter the edges connecting two different types of cells. Additionally, we have heterogeneous edges named “gene_expressed_by_cell” and “cell_express_gene” in opposite directions to indicate a gene is expressed by a cell and a cell expresses a gene.

### Architecture of STAMapper

STAMapper consists of two parts, i.e., a heterogeneous graph encoder and a heterogeneous graph attention classifier (Fig. 1a). STAMapper updates parameters on heterogeneous graphs according to the message-passing mechanism, where the same edge type shares the same parameters [27]. We aim to learn these edge parameters.

#### Encoder

The encoder is based on the architecture of the heterogeneous graph, where we take the expression of the union of DEGs from different clusters as the input for each cell node. Suppose we have $n$ cells in the scRNA-seq dataset and $m$ cells in the scST dataset. Here, we denote ${{\varvec{x}}}_{i}^{c}={\left({x}_{i1}^{c},{x}_{i2}^{c},\dots ,{x}_{ip}^{c}\right)}^{T}$ as the input for cell node $i$ $(i\in \left\{1,\dots ,n,\dots ,N=m+n\right\}),$ where ${x}_{ij}^{c}$ denotes the normalized expression for gene $j$ $(j\in \{{1,\!2},\dots ,p\})$ in cell $i$. For the cell node $i$ the initial embedding is calculated as follows:

$${{\varvec{h}}}_{i}^{c(0)}=LN\left(\sigma \left({{\varvec{w}}}_{c}^{\left(0\right)}{{\varvec{x}}}_{i}^{c}+{{\varvec{b}}}_{c}^{\left(0\right)}\right)\right),$$

where $LN$ denotes the layer normalization [71], $\sigma$ is the nonlinear activation function, ${{\varvec{w}}}_{c}^{(0)}$ denotes the learnable parameters for the edge type “cell_self_loop” in the initial layer, ${{\varvec{b}}}_{c}^{(0)}$ denotes the learnable bias for cell nodes. We used the intersection of selected genes (DEGs and HVGs) between scRNA-seq and scST datasets as the gene nodes. Suppose we have $s$ gene nodes, the initial embedding for gene node $k$ is as follows:

$${{\varvec{h}}}_{k}^{g(0)}=LN\left(\sigma \left(\sum_{i\in {N}_{c}^{gk}}\frac{1}{\left|{N}_{c}^{gk}\right|}{{\varvec{w}}}_{cg}^{\left(0\right)}{{\varvec{x}}}_{i}^{c}+{{\varvec{b}}}_{g}^{\left(0\right)}\right)\right),$$

where ${N}_{c}^{gk}$ is a set containing the cell nodes which are neighbors of gene node $k$ where ${|N}_{c}^{gk}|$ denotes the number of cells in this set. ${{\varvec{w}}}_{cg}^{(0)}$ denotes the learnable parameters for edge type “cell_express_gene” for the initial layer, ${{\varvec{b}}}_{g}^{(0)}$ denotes the learnable bias for gene nodes.

For the $l$ th $(1\le l\le L)$ hidden layer, the embedding for cell node $i$ is:

$${{\varvec{h}}}_{i}^{c(l)}=LN\left(\sigma \left(\sum_{r\in RE{T}_{c}}\sum_{j\in {N}_{r}^{ci}}\frac{1}{\left|{N}_{r}^{ci}\right|}{{\varvec{w}}}_{r}^{\left(l\right)}{{\varvec{h}}}_{j}^{r\left(l-1\right)}+{{{\varvec{w}}}_{c}^{\left(l\right)}{\varvec{h}}}_{i}^{c\left(l-1\right)}+{{\varvec{b}}}_{c}^{\left(l\right)}\right)\right),$$

where $RE{T}_{c}$ denotes a set of edge types connected to cell node $c$, ${N}_{r}^{ci}$ denotes a set containing the cell/gene nodes that are neighbors of cell node $i$ according to edge type $r$, ${{\varvec{w}}}_{r}^{(l)}$ denotes the learnable parameters for edge type $r$, ${{\varvec{h}}}_{i}^{r(l-1)}$ denotes the embedding for the $i$ th cell/gene node (adaptive to the edge type $r$) in the previous layer. The embedding for gene node $k$ in the $l$-th hidden layer is:

$${{\varvec{h}}}_{k}^{g(l)}=LN\left(\sigma \left(\sum_{r\in RE{T}_{g}}\sum_{j\in {N}_{r}^{gk}}\frac{1}{\left|{N}_{r}^{gk}\right|}{{\varvec{w}}}_{r}^{\left(l\right)}{{\varvec{h}}}_{j}^{r\left(l-1\right)}+{{{\varvec{w}}}_{g}^{\left(l\right)}{\varvec{h}}}_{k}^{g\left(l-1\right)}+{{\varvec{b}}}_{g}^{\left(l\right)}\right)\right),$$

$RE{T}_{g}$ denotes a set of edge types connecting to gene node $g$, ${N}_{r}^{gk}$ denotes a set containing the cell nodes that are neighbors of gene node $k$ according to edge type $r$, ${{\varvec{w}}}_{r}^{(l)}$ denotes the learnable parameters for edge type $r$, ${{\varvec{h}}}_{j}^{r(l-1)}$ denotes the embedding for the $j$ th cell/gene node (adaptive to the edge type $r$) in the previous layer.

#### Classifier

To further utilize the information from genes associated with cell classification, we employed an attention mechanism [27] in the heterogeneous graph classifier. Specifically, the attention weight of the classifier from gene node $j$ to cell node $i$ is:

$${e}_{ij}=leakyReLU\left({\mathbf{v}}^{{\varvec{T}}}\left[{\mathbf{w}}_{c}{{\varvec{h}}}_{i}^{c(L)}||{\mathbf{w}}_{g}{{\varvec{h}}}_{j}^{g(L)}\right]\right),$$

where $\mathbf{v}$ is a learnable weight vector, $\left[{\mathbf{w}}_{c}{{\varvec{h}}}_{i}^{c(L)}||{\mathbf{w}}_{g}{{\varvec{h}}}_{j}^{g(L)}\right]$ denotes concatenation of ${\mathbf{w}}_{c}{{\varvec{h}}}_{i}^{c(L)}$ and ${\mathbf{w}}_{g}{{\varvec{h}}}_{j}^{g(L)}$. The weight is further normalized as follows:

$$at{t}_{ij}=\frac{\textrm{exp}({e}_{ij})}{\sum_{k\in {N}_{g}^{ci}}\textrm{exp}\left({e}_{ik}\right)},$$

where ${N}_{g}^{ci}$ is a set containing the gene nodes that are neighbors of cell node $i$, determined by edge “gene_expressed_by_cell.” The normalized attention weight $at{t}_{ij}$ reflects the importance of gene $j$ in determining the classification of cell $i$. Given a gene and a specific cell type, we aggregate the normalized attention weights from that gene to all cells belonging to the cell type and compute their average as the cell type’s normalized attention weights. This value represents the heatmap intensity shown in Fig. 5i, reflecting the overall importance of the gene for that particular cell type determined by STAMapper. Then the output logits of the classifier are:

$${{\varvec{h}}}_{i}^{c(out)}=\sum_{k\in {N}_{g}^{ci}}at{t}_{ij}{\mathbf{w}}_{g}{{\varvec{h}}}_{j}^{g(L)}+{{\varvec{b}}}^{(out)}.$$

Then we apply $softmax$ function over logits coordinately:

$${{\varvec{Y}}}^{\prime}=softmax\left({{\varvec{H}}}^{c\left(out\right)}\right),$$

where ${{\varvec{H}}}^{c\left(out\right)}=({{\varvec{h}}}_{1}^{c\left(out\right)},{{\varvec{h}}}_{2}^{c\left(out\right)},\dots ,{{\varvec{h}}}_{N}^{c\left(out\right)})$, ${{\varvec{Y}}}_{i}^{\prime}\in {R}^{N\times C}$. Here, $N$ denotes the total number of cells, ${\varvec{C}}$ denotes the number of cell types, ${{\varvec{Y}}}_{ij}^{\prime}$ denotes the predicted probabilities of cell type $j$ for the $i$ th cell.

#### Loss function

We modified the cross-entropy loss as the classification loss. Suppose ${{\varvec{y}}}_{sc}$ is the manual annotation of cells from the scRNA-seq dataset. We first use a weighted cross-entropy loss as follows:

$${L}_{1}\left({{\varvec{y}}}_{sc},{{\varvec{y}}}_{sc}^{\boldsymbol{^{\prime}}}\right)=-\frac{1}{m}\sum_{i=1}^{m}\sum_{j=1}^{C}{w}_{j}{y}_{ij}log{{y}{\prime}}_{ij}+\lambda \sum_{k}{\theta }_{k}^{2},$$

where ${w}_{j}\propto \frac{1}{\sqrt{{m}_{c}}}$ denotes the weight for cell-type $j$, ${m}_{c}$ denotes the number of cells in $c$-th cell type, $\sum_{k}{\theta }_{k}^{2}$ denotes the sum of the squares of all model parameters penalizing the complexity of the model to prevent overfitting, and $\lambda$ is set as 0.01 by default. We applied the label smoothing technique to reduce the model overconfidence [28]. The overall training loss is:

$$L\left({{\varvec{y}}}_{sc},{{\varvec{y}}}_{sc}^{\boldsymbol{^{\prime}}}\right)=\left(1-\epsilon \right){L}_{1}\left({{\varvec{y}}}_{sc},{{\varvec{y}}}_{sc}^{\boldsymbol{^{\prime}}}\right)+\frac{\epsilon}{Cm}\sum_{i=1}^{m}\sum_{j=1}^{C}\log({{y}^{\prime}}_{ij}),$$

the default value of $\epsilon$ is 0.1.

#### Training process

In all experiments, we set the encoder of STAMapper as a two-layer heterogeneous graph neural network with 512 hidden units for all types of edges and nodes across all hidden layers. We apply the Adam optimizer [72] with a learning rate of 5e-3 to optimize all the parameters. We adopt LeakyReLU [73] as the activation function with a negative slope set as 0.05. We set the number of iterations as 1000 by default and use the same checkpoint selection strategy as a recently published method [74].

### Unknown cells detection

We detected unknown cells based on two criteria: (i) cells in the scST data with predicted probability less than the median value; and (ii) cell types with distance from the scST data to scRNA-seq data larger than a user-defined threshold (default 4). The distance was calculated based on the cell embeddings provided by STAMapper, after reducing to 50 dimensions using PCA. Specifically, the distance for a given cell type from the scST data to scRNA-seq data is defined as the average distance of each cell in the scST data to its five nearest neighbors in the scRNA-seq data.

### Gene module extraction and enrichment analysis

We performed the Leiden community detection algorithm [29] on the gene embeddings from the last layer of STAMapper and defined the detected clusters as gene modules. We performed the GO enrichment analysis by the R package clusterProfiler [75] with the biological process ontologies and a *p* value cutoff of 0.01 to identify significantly enriched terms.

### Evaluation

To assess the performance of cell-type annotation, we employed accuracy, macro F1 score, and weighted F1 score as evaluation metrics. Accuracy is defined as the proportion of correctly predicted cells among all cells. In addition, we employed macro F1 and Weighted F1. To define macro F1 score and weighted F1 score, we first define precision and recall for each cell type:

precision $$=\frac{\textrm{TP}}{\textrm{TP}+\textrm{FP}}$$, recall $$=\frac{\textrm{TP}}{\textrm{TP}+\textrm{FN}}$$,

respectively, where TP, FP, and FN represent the number of true positives, false positives, and false negatives, respectively. The F1 score is the harmonic mean of precision and recall, while the Macro F1 is defined as the average of class-wise F1 score,

Macro F1 score $$=\frac{1}{\textrm{C}}{\sum }_{\mathrm{c}=1}^{\mathrm{C}}{\mathrm{F}}_{1}^{\left(\mathrm{c}\right)}$$,

where ${\mathrm{F}}_{1}^{\left(\mathrm{c}\right)}$ represents the F1 score for cell type $\mathrm{c}$, and $\mathrm{C}$ denotes the number of all cell types. The weighted F1 considers the proportion of each cell type,

Weighted F1 score $$={\sum }_{\mathrm{c}=1}^{\mathrm{C}}\frac{{\textrm{m}}_{\textrm{c}}}{\textrm{m}}\times {\mathrm{F}}_{1}^{\left(\mathrm{c}\right)}$$,

where ${\mathrm{m}}_{\mathrm{c}}/\mathrm{m}$ represents the proportion of cells of type $\mathrm{c}$ in all cells.

### Benchmarking cell-type annotation

We performed scANVI [23] with the Python package scVI, referring to the section “Integration and label transfer with Tabula Muris” from its documentation (https://docs.scvi-tools.org/en/stable/tutorials/notebooks/scrna/tabula_muris.html.). We performed RCTD using the R package spacexr [24] with default workflow (https://raw.githack.com/dmcable/spacexr/master/vignettes/spatial-transcriptomics.html). We performed Tangram [25] with the Python package with cluster mode (https://tangram-sc.readthedocs.io/en/latest/tutorial_sq_link.html). We performed SeuratV4 [32] with the R package Seurat, referring to the section “Mapping and annotating query datasets” from its documentation (https://satijalab.org/seurat/articles/integration_mapping). We performed CellTrek [31] with the R package CellTrek, referring to its tutorial (https://github.com/navinlabcode/CellTrek). We set “intp = F” for the function celltrek () with all other parameters as default. After projecting the scRNA-seq cells onto the scST coordinates, we inspected every cell in the scST dataset. If one or more scRNA-seq cells were mapped to a given spatial location, we assigned that location the cell type corresponding to the most prevalent mapped cell population. For locations without any mapped scRNA-seq cells, we identified the five nearest neighboring scRNA-seq cells and assigned the cell type by majority vote among those neighbors. We performed CAESAR [69] with the R package CAESAR.Suite, referring to its tutorial (https://xiaozhangryy.github.io/CAESAR.Suite/articles/STMOB.html). For scST datasets with less than 2000 genes, we set the overlap.max of the function marker.select() as 4 to obtain better annotation results. Additionally, for some datasets, the algorithm reported the default radius.upper value of 400 in the CAESAR.coembedding() function was too small to identify spatial neighbors, so we increased it to 800.

### Spatial clustering with STAGATE

We performed STAGATE [58] on the Slide-tags cortex data and followed a standard workflow for data preprocessing with a rad_cutoff as 200 to construct the spatial network (https://stagate.readthedocs.io/en/latest/T3_Slide-seqV2.html).

### Visualization of the UMAP plot

The UMAP embeddings presented in our study were computed and visualized using the Scanpy package. Specifically, the neighborhood graph was generated with scanpy.pp.neighbors() with n_neighbors = 10, n_pcs = 40, based on the PCA-reduced representation of the data. We then used scanpy.tl.umap() to embed the neighborhood graph using UMAP and visualized the results with scanpy.pl.umap(). All other parameters were set to their default values.

## Supplementary Information

Additional file 1: Figures S1-S6, Tables S1-S2. Supplementary figures that complement the analyses in the main text, and tables detailing the collected datasets.

Additional file 2: Tables S3. Running time and peak memory cost of STAMapper and competing methods.

## Data Availability

All datasets analyzed in this study are publicly available. The raw datasets are available from the following studies: Dataset 1–4 (mouse prefrontal cortex):STARmap [15, 76] https://github.com/weallen/STARmap/tree/master; 10x Chromium [15, 77], adult samples from GSE124952 in the GEO database. Dataset 5–9 (mouse visual cortex): STARmap [15, 76], https://github.com/weallen/STARmap/tree/master; Smart-seq [78, 79], https://portal.brain-map.org/atlases-and-data/rnaseq/mouse-v1-and-alm-smart-seq. Dataset 10 (mouse visual cortex): seqFISH + [13, 80], https://github.com/CaiGroup/seqFISH-PLUS; Smart-seq [78, 79], https://portal.brain-map.org/atlases-and-data/rnaseq/mouse-v1-and-alm-smart-seq. Dataset 11 (mouse somatosensory cortex): osmFISH [79, 81], https://github.com/drieslab/spatial-datasets/tree/master/data/ 2018_osmFISH_SScortex/raw_data; STRT/C1 [82, 83], GSE60361 in the GEO database. Dataset 12–23 (mouse primary motor cortex): MERFISH [11, 84], https://knowledge.brain-map.org/data/L3GYGFMDJCG0GUEE3QG/; 10x Chromium [85, 86], https://data.nemoarchive.org/biccn/lab/zeng/transcriptome/scell/10x_v3/mouse/processed/analysis/10X_cells_v3_AIBS/. Dataset 24–33 (mouse retina): MERFISH [33, 87], https://zenodo.org/records/8144355; 10x Chromium [34, 88], GSE135406 in the GEO database. Dataset 34 (mouse kidney): MERFISH [26, 89], https://figshare.com/projects/ MERFISH_mouse_comparison_study/134213; 10x Chromium [26, 89], https://figshare.com/articles/dataset/SingleCellData_raw_/19310675. Dataset 35 (mouse liver): MERFISH [26, 89], https://figshare.com/projects/ MERFISH_mouse_comparison_study/134213; 10X Chromium [26, 89], https://figshare.com/articles/dataset/SingleCellData_raw_/19310675. Dataset 36–71 (mouse hypothalamic preoptic region): MERFISH [22, 90], https://datadryad.org/stash/dataset/10.5061/dryad.8t8s248; Droplet-microfluidic [22, 91], GSE113576 in the GEO database. Dataset 72–77 (mouse gastrulation):osmFISH, seqFISH [81, 92], https://crukci.shinyapps.io/mousegastrulation2018/; 10x Chromium [81, 93], https://bioconductor.org/packages/devel/data/experiment/vignettes/MouseGastrulationData/inst/doc/MouseGastrulationData.html. Dataset 78–79 (mouse prefrontal cortex): STARmap PLUS [16, 94], https://zenodo.org/records/7332091; 10x Chromium [15, 77], GSE124952 in the GEO database. Dataset 80 (human prefrontal cortex): Slide-tags [18, 95], https://singlecell.broadinstitute.org/single_cell/study/SCP2167/slide-tags-snrna-seq-on-human-prefrontal-cortex#study-download; 10x Chromium [52, 96], GSE168408 in the GEO database. Dataset 81 (human liver cancer): NanoString [17, 97], https://nanostring.com/resources/liver-cancer-raw-data-files-cosmx-smi-human-liver-ffpe-dataset/; 10x Chromium [39, 97], GSE149614 in the GEO database.

## Code availability

STAMapper is implemented in Python, it is available on GitHub under an open-source MIT license (https://github.com/zhanglabtools/STAMapper) [98] and have been deposited in Zenodo with a permanent DOI (https://zenodo.org/records/17007394) [99].
