# Supplementary material for: High-precision cell-type mapping and annotation of single-cell spatial transcriptomics with STAMapper
Source: Genome Biol. 2025 Oct 7;26:342. doi: 10.1186/s13059-025-03773-6 (PMC12502291; doi:10.1186/s13059-025-03773-6)
Supplement: Supplementary file 1 — Additional file 1: Figures S1-S6, Tables S1-S2. Supplementary figures that complement the analyses in the main text, and tables detailing the collected datasets. [file 13059_2025_3773_MOESM1_ESM.pdf]

Supplementary Materials for

High-precision cell-type mapping and annotation of single-cell spatial transcriptomics with STAMapper

Qunlun Shen[1,3], Kangning Dong[2,3], Shuqin Zhang[1,4,5*] and Shihua Zhang[3,6,7*]

[1]School of Mathematical Sciences, Fudan University, Shanghai, 200433, China.
[2]School of Mathematics, Renmin University of China, Beijing 100872, China.
[3]State Key Laboratory of Mathematical Sciences, Academy of Mathematics and Systems Science, Chinese Academy of Sciences, Beijing 100190, China;
[4]Key Laboratory of Mathematics for Nonlinear Science, Fudan University, Ministry of Education, Shanghai, 200433, China.
[5]Shanghai Key Laboratory for Contemporary Applied Mathematics, Fudan University, Shanghai, 200433, China.
[6]School of Mathematical Sciences, University of Chinese Academy of Sciences, Beijing 100049, China;
[7]Key Laboratory of Systems Health Science of Zhejiang Province, School of Life Science, Hangzhou Institute for Advanced Study, University of Chinese Academy of Sciences, Chinese Academy of Sciences, Hangzhou 310024, China;

*To whom correspondence should be addressed. Shuqin Zhang, Tel: 86-21-65647484; Email: zhangs@fudan.edu.cn. Shihua Zhang, Tel: 86-10-82541360; Email: zsh@amss.ac.cn.

Supplementary note 1:
A recent proposed method, CAESAR[1], integrates histology images and spatial location information into a low-dimensional space to characterize the gene-cell relationship. We compared the cell-type annotation performance of STAMapper with CAESAR with the histology image feature-extraction module disabled. The annotation performance of STAMapper was better in terms of accuracy, macro F1 score, and weighted F1 score (**Additional file 1: Fig. S6g**).

Supplementary Figures

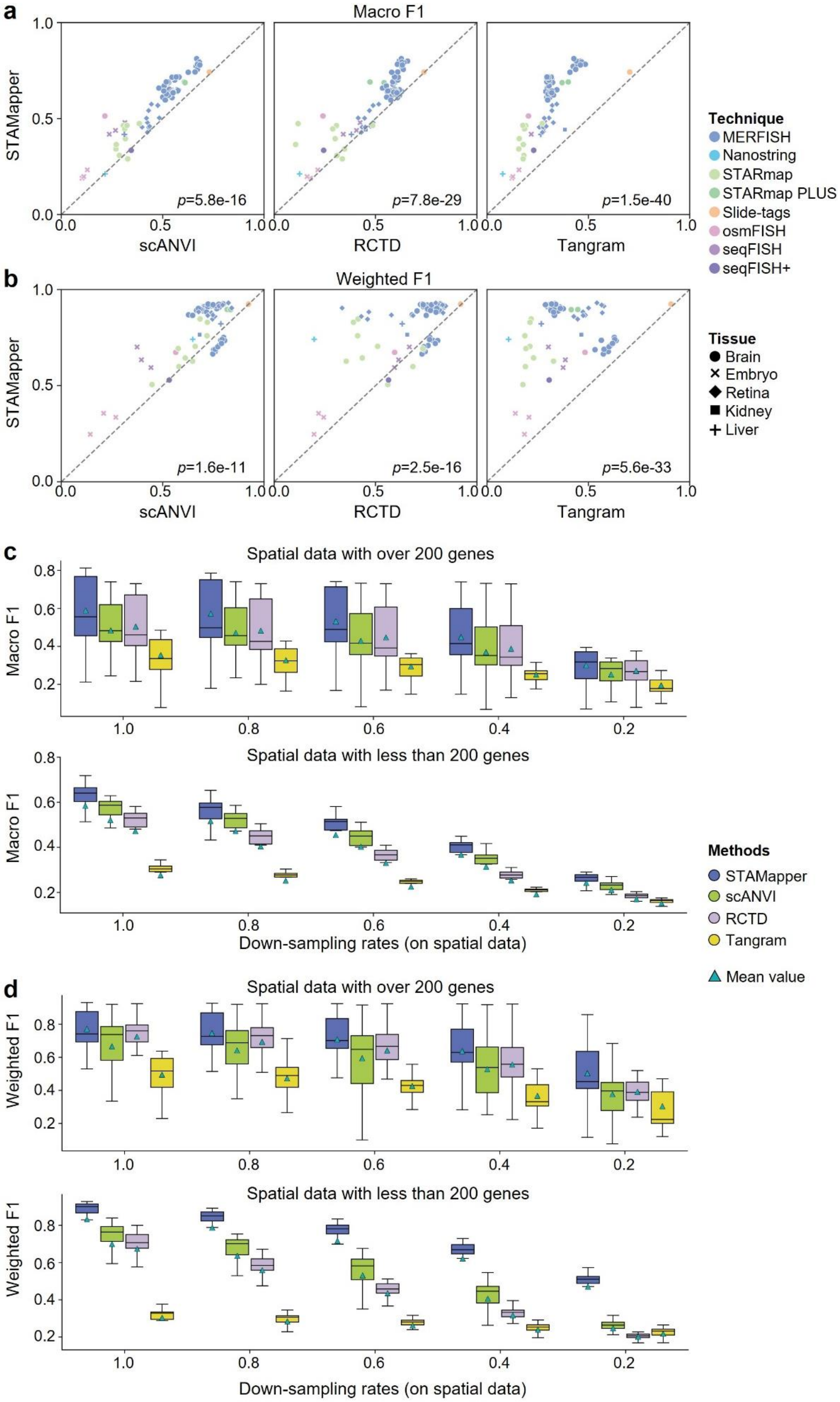

Fig. S1 (continue)

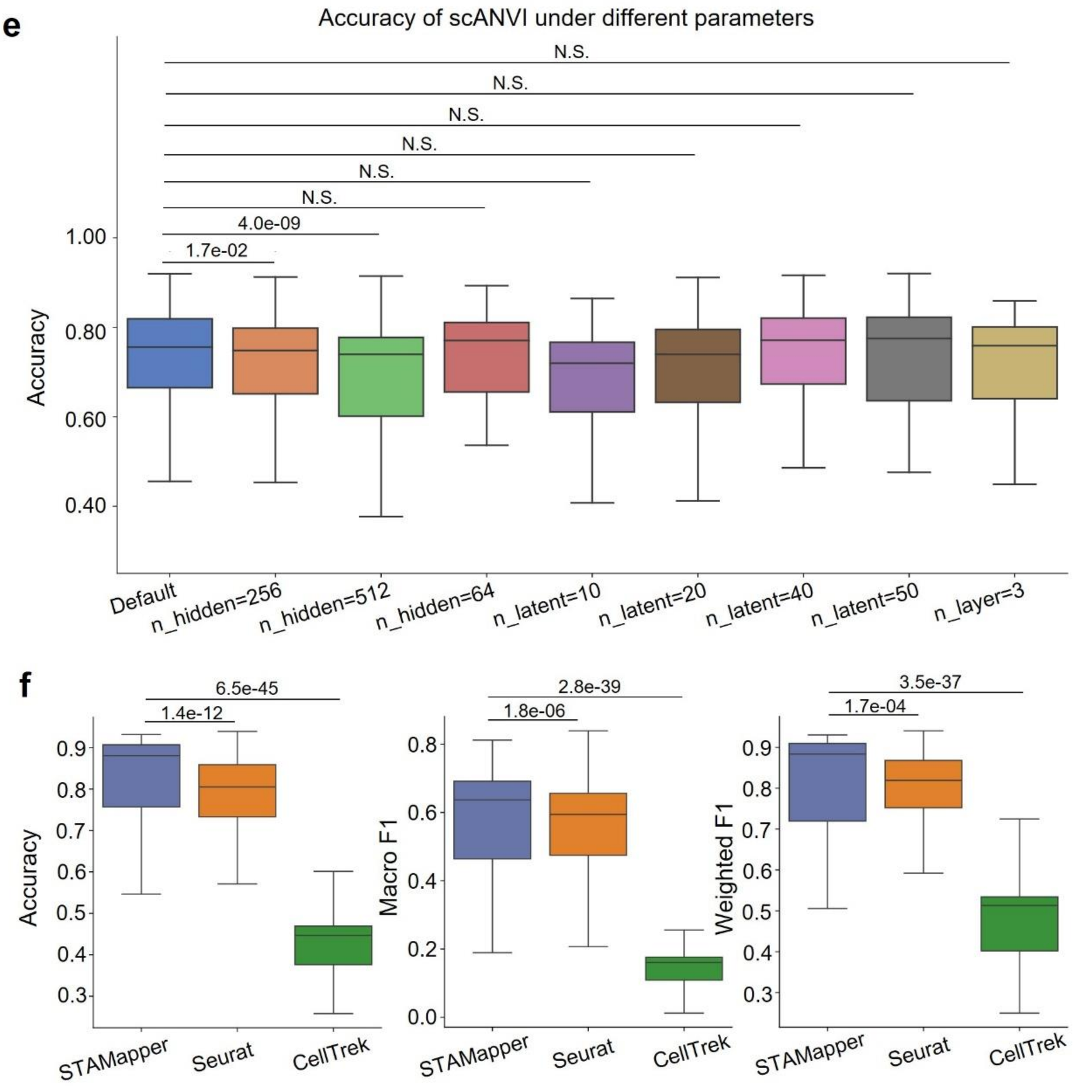

Fig. S1. a and **b.** Performance comparison of STAMapper and three other methods in terms of macro F1 score **(a)** and weighted F1 score **(b)** on the 81 pairs of scRNA-seq and scST datasets. *p* values were calculated using a paired t-test. **c** and **d.** Performance comparison of the macro F1 score **(c)** and weighted F1 score **(d)** of STAMapper and three other methods on different down-sampling rates (1.0, 0.8, 0.6, 0.4, 0.2) for read counts, where the down-sampling rate of 1.0 means the raw data. The upper panel displays the scST datasets with more than 200 genes for sequencing, while the lower panel corresponds to datasets with fewer than 200 genes. **e.** The accuracy of scANVI under different parameters, with the default settings being n_hidden=128, n_layers=2, and n_latent=30. **f.** Performance comparison of STAMapper, Seurat, and CellTrek. *P*-values were calculated using paired t-tests.

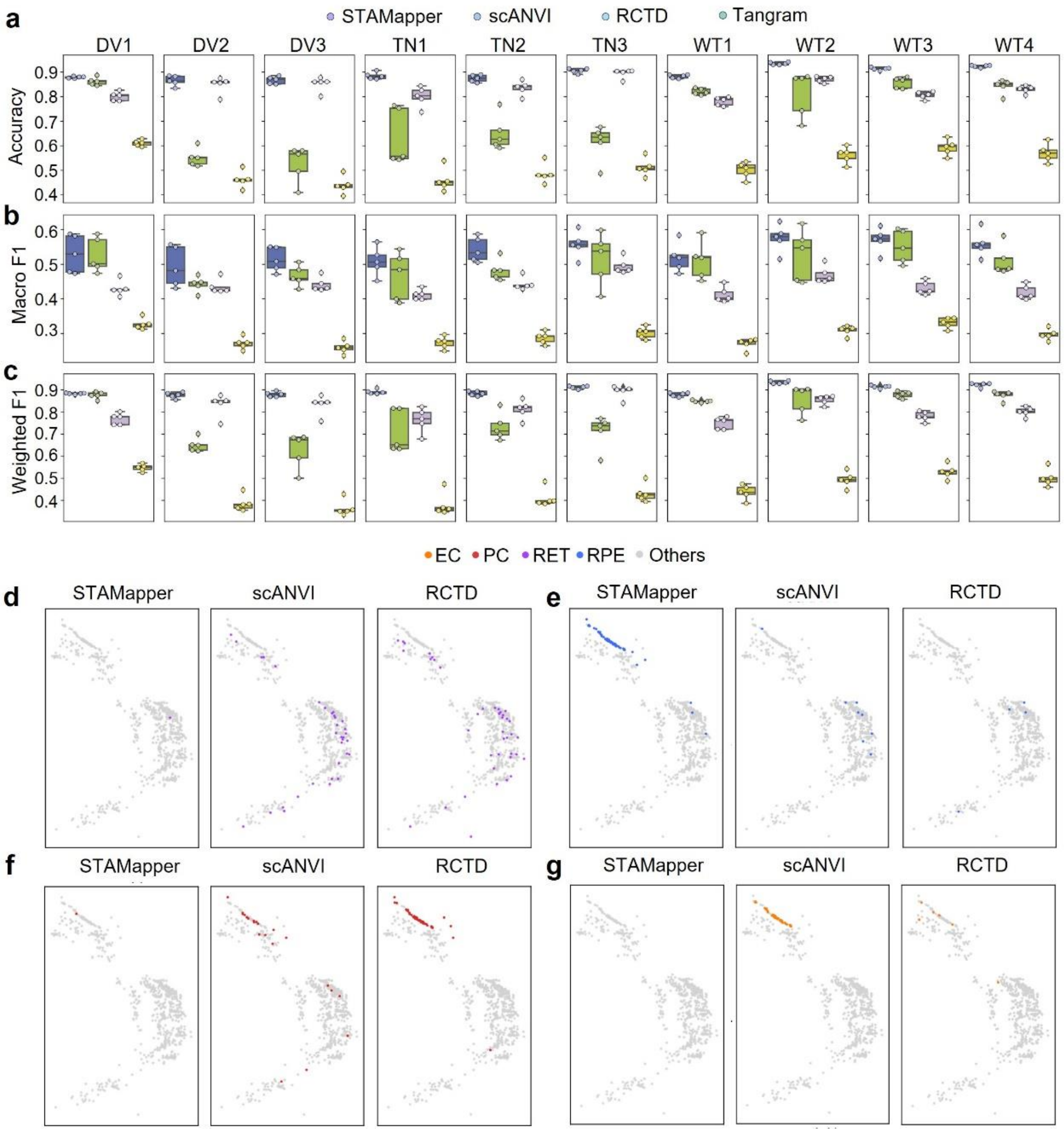

Fig. S2 (continue)

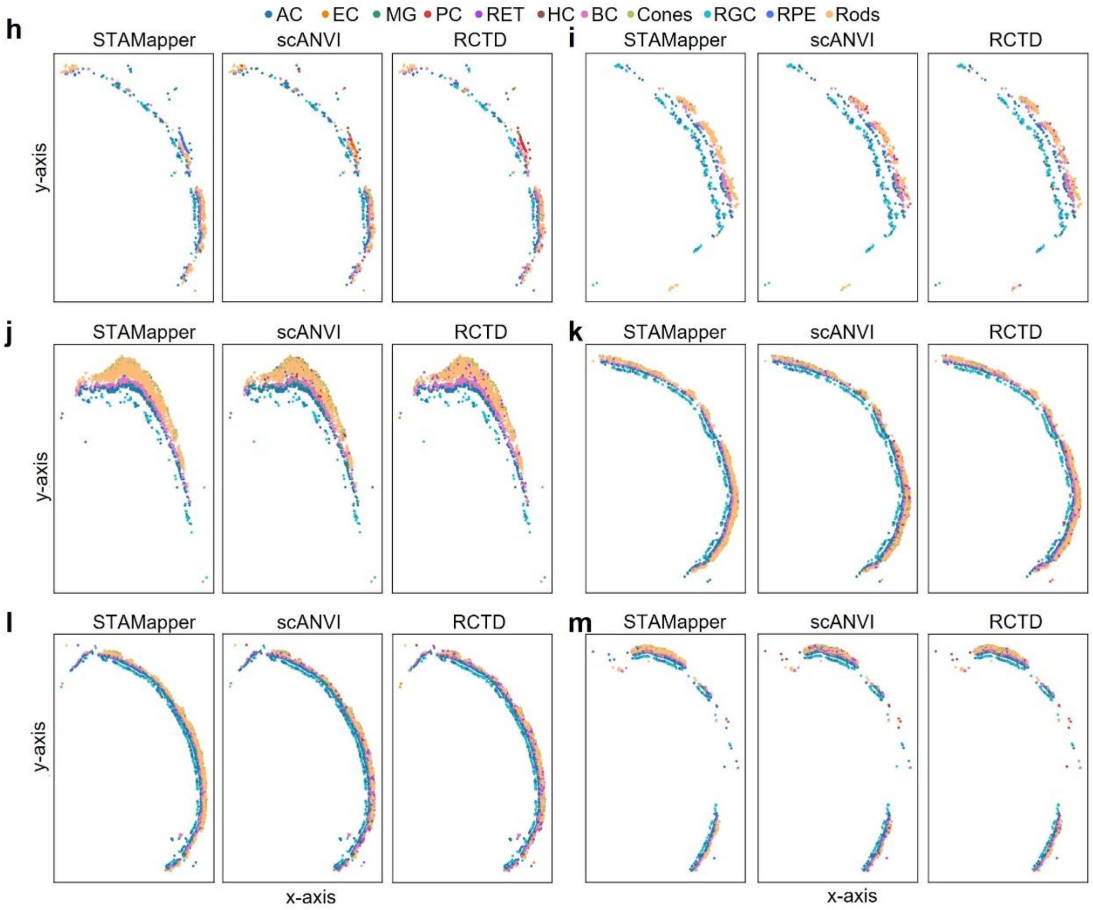

Fig. S2. a and **c.** Box plots showing the accuracy **(a)**, macro F1 score **(b)**, and weighted F1 score **(c)** on the 50 paired datasets (5 scRNA-seq datasets and 10 scST datasets). Each column corresponds to a scST dataset. **d-g.** Spatial organization of EC, PC, RET, and RPE in the scST data, corresponding to **Fig. 3e**. **h-m.** Spatial organization of the remaining six slices from the scST data corresponding to **Fig. 2b**, where cells are colored by the annotation by STAMapper, scANVI, and RCTD, respectively.

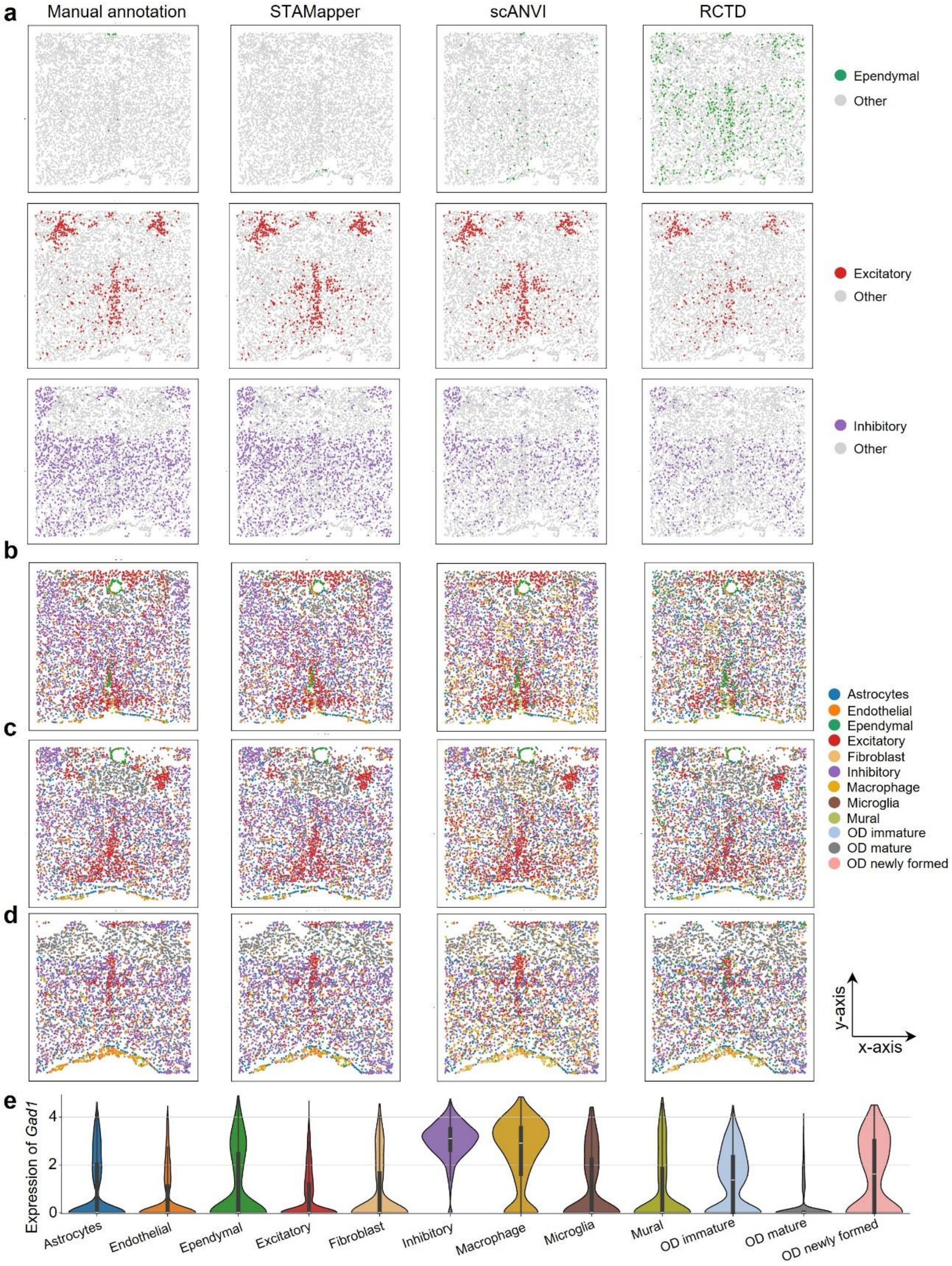

Fig. S3 (continue)

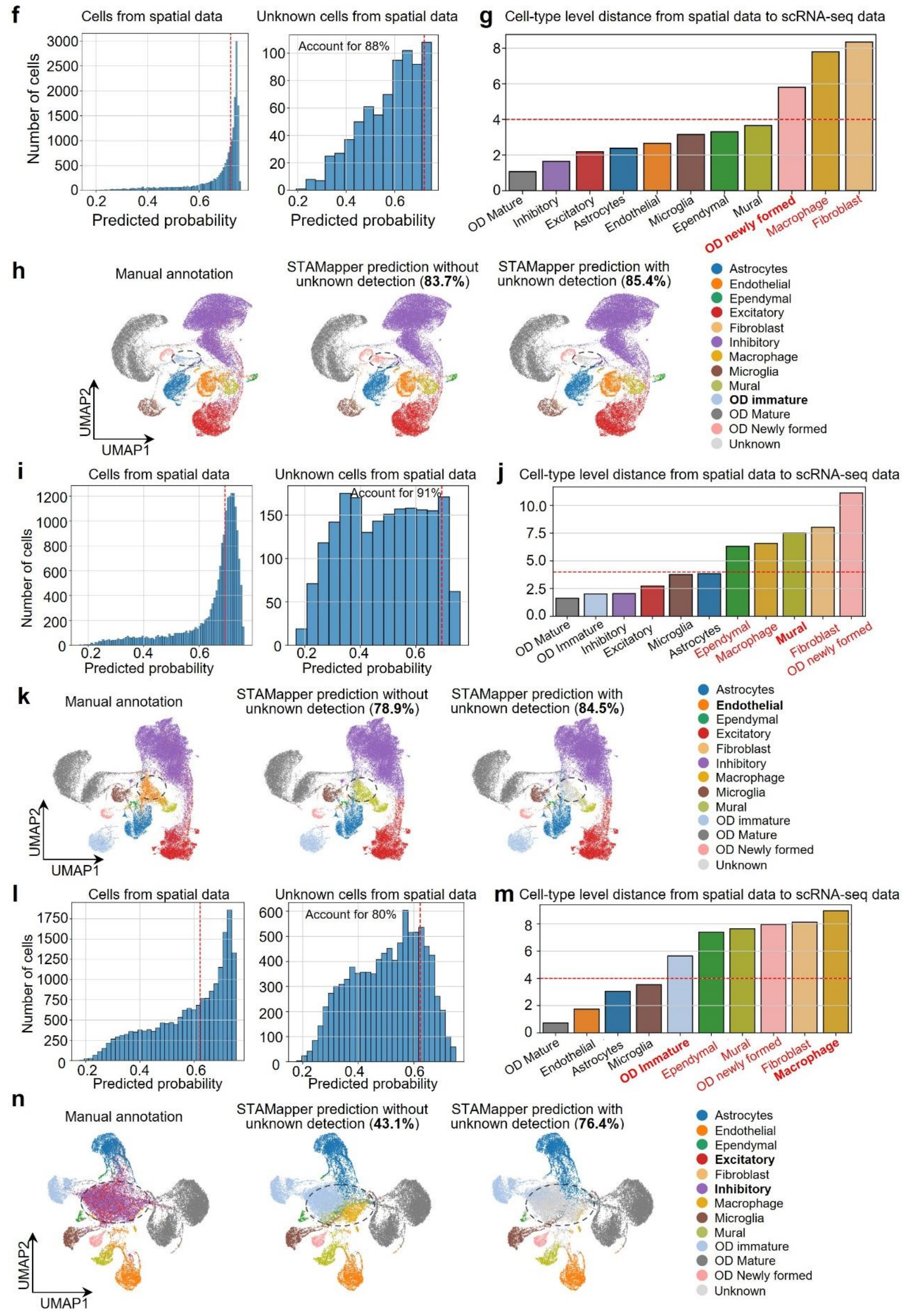

Fig. S3 (continue)

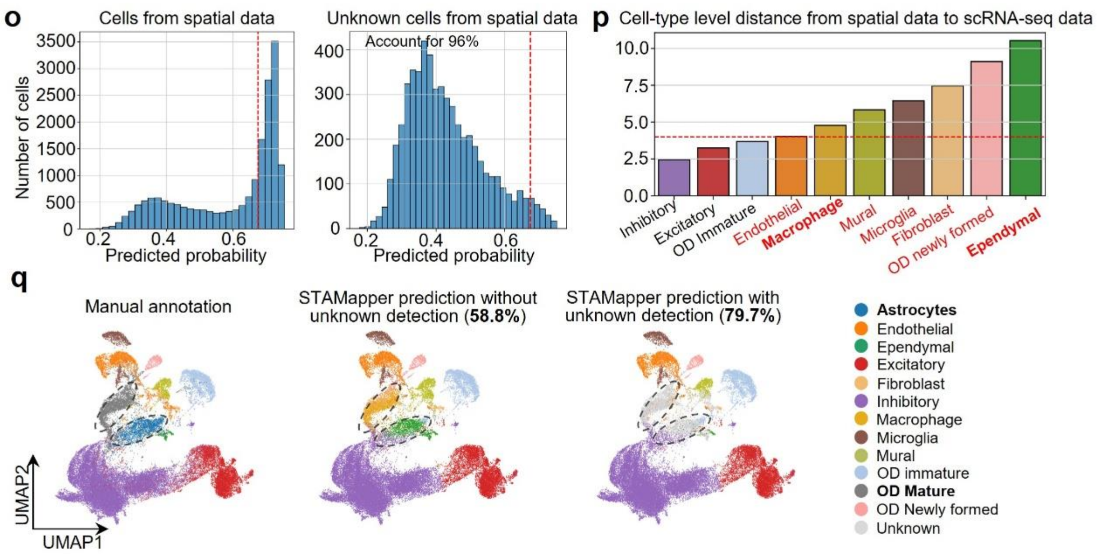

Fig. S3. a. Spatial organization of Ependymal, Excitatory, and Inhibitory cells from the mouse hypothalamic dataset corresponding to **Fig. 4b**. Cells are colored by the manual annotation and predictions of STAMapper, scANVI, and RCTD, respectively. **b-d**, Spatial organization of the remaining three slices from the mouse hypothalamic data corresponding to **Fig. 4b**. Cells are colored based on the manual annotation and prediction by STAMapper, scANVI, and RCTD, respectively. **e.** Expression levels of *Gad1* (a marker of inhibitory cells) across different cell types (annotated by scANVI). **f, i, l, o.** The predicted probability of STAMapper for each cell from the scST data (left panel), and unknown cells from the scST data (right panel). **g, j, m, p.** Cell-type level distance from the scST data to the scRNA-seq data on the embedding learned by STAMapper. Bold font indicates unknown cells were predicted as this specific cell type, and red font denotes cell types present in single-cell data but not annotated in the scST data by manual annotation. **h, k, n, q.** UMAP plots showing the co-embedding of the scRNA-seq and scST data learned by STAMapper, where cells are colored by manual annotation, STAMapper prediction without unknown detection, and STAMapper prediction with unknown detection, respectively. The percentages in parentheses represent the predicted accuracy. Bold font in legend indicates cell types that were removed from the scRNA-seq data.

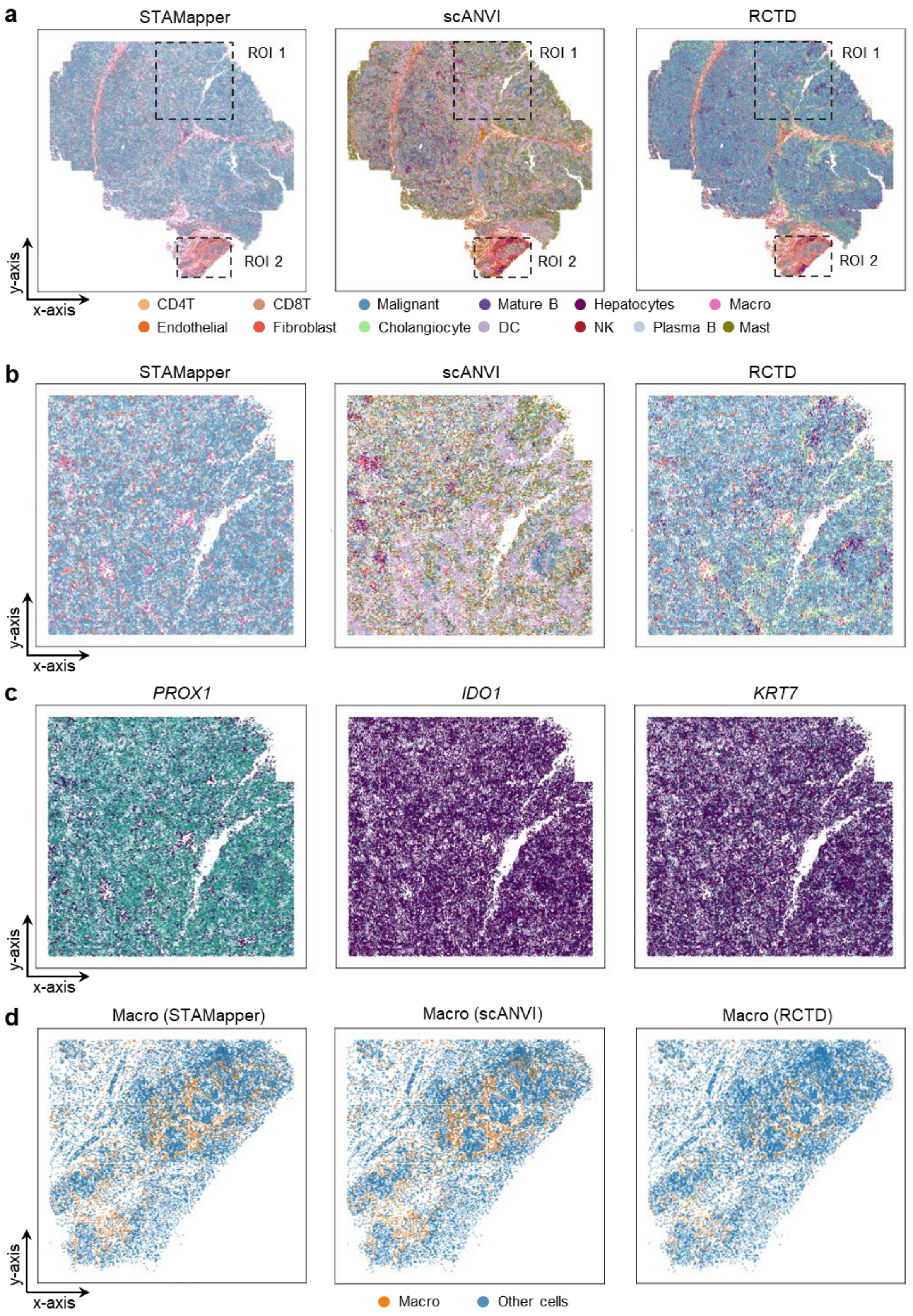

Fig. S4. a. Spatial organization of the Nanostring HCC dataset corresponding to **Fig. 5a**, **b.** Spatial organization of ROI 1, where cells are colored based on the annotation by STAMapper, RCTD, and scANVI, respectively. **c.** The normalized maker expression on ROI 1. **d.** The distribution of Macro in ROI 2.

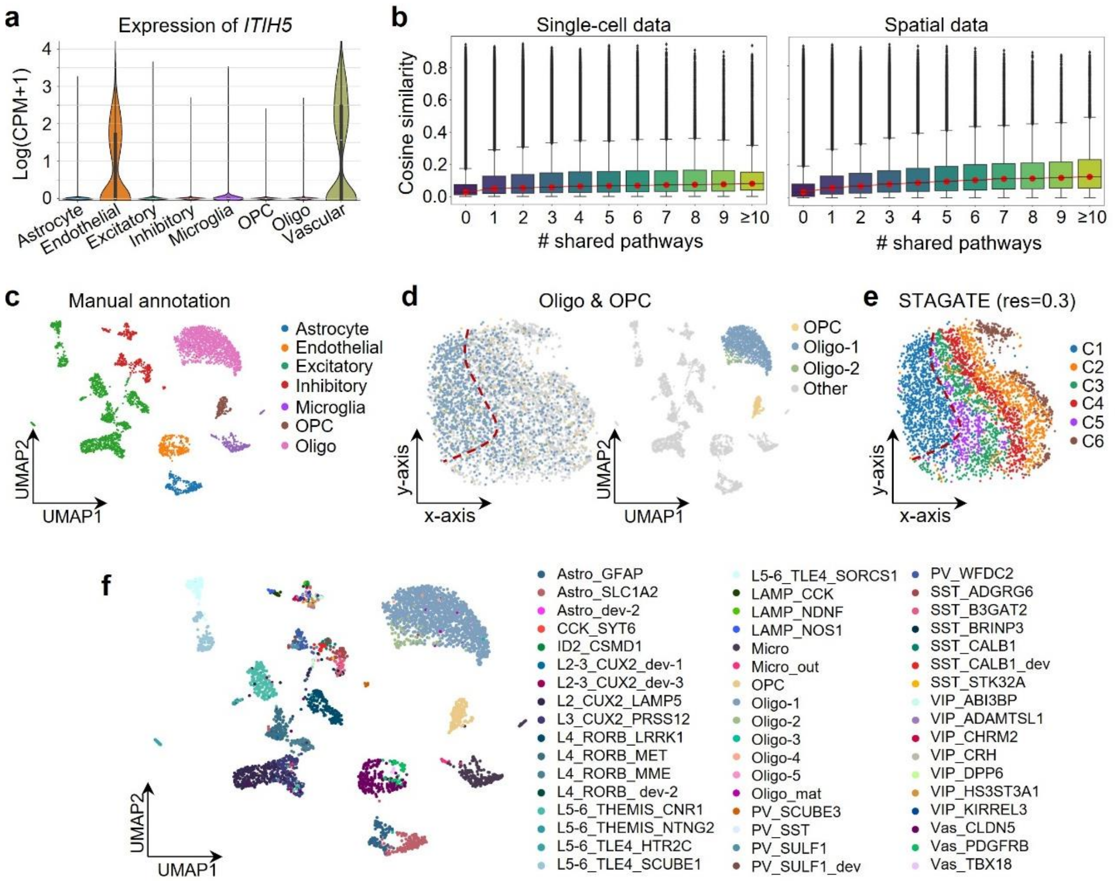

Fig. S5. a. Violin plot showing the expression of *ITIH5* across different cell types. **b.** Boxplots showing the cosine similarity between the expression vector of gene pairs from the scRNA-seq data (left panel) and scST data (right panel), and the gene pairs are grouped by the number of shared pathways. **c.** UMAP plots of the spatial data and cells colored by manual annotation. **d.** Spatial organization and UMAP plot of Oligo & OPC subtypes (predicted by STAMapper) from the scST data, and subtypes with more than 20 cells are shown. **e.** Spatial organization of cells from the scST data, where cells are clustered by STAGATE with resolution=0.3. **f.** UMAP plots showing the distribution of cell subtypes on the scST data predicted by STAMapper.

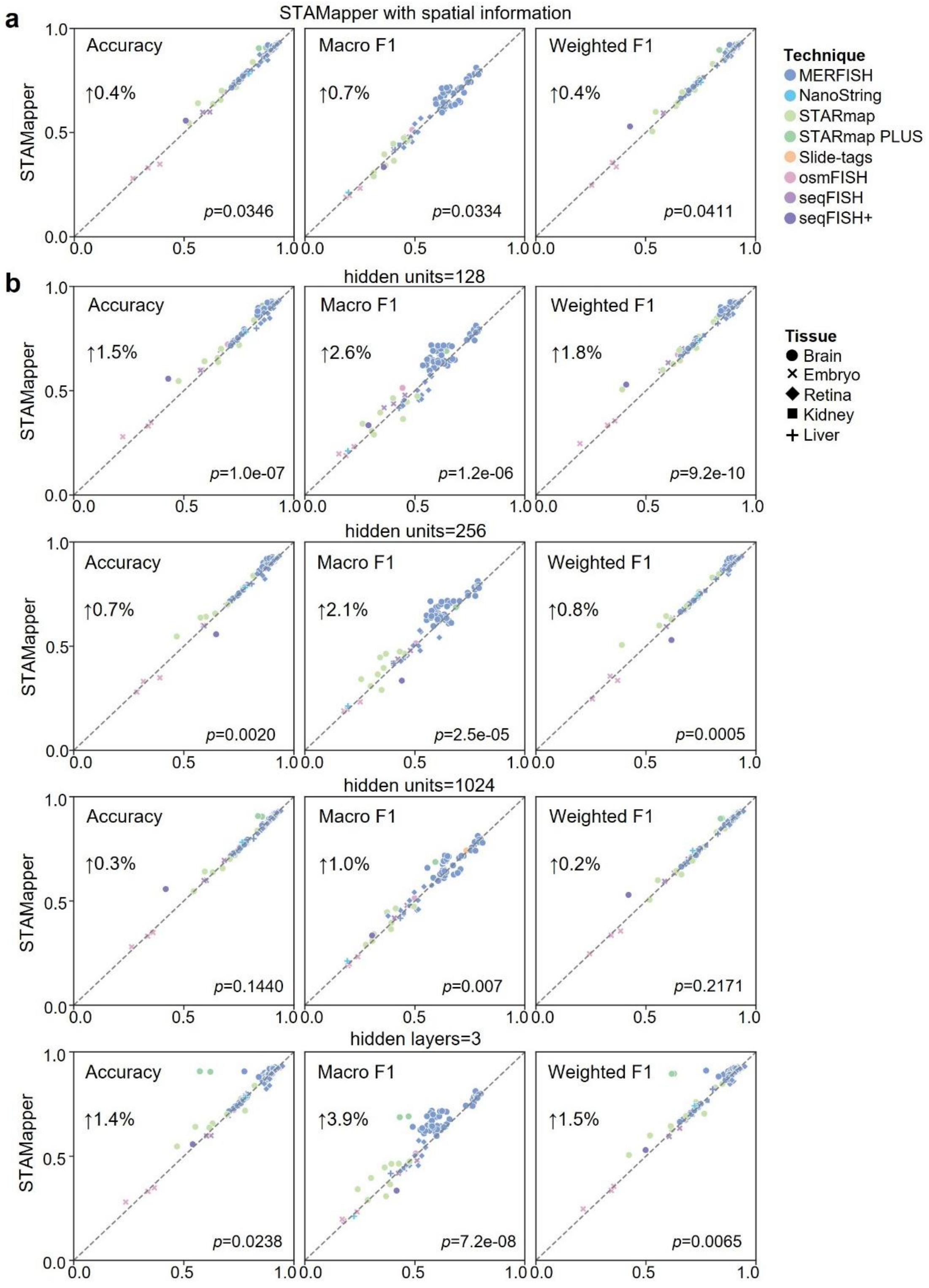

Fig. S6 (continue)

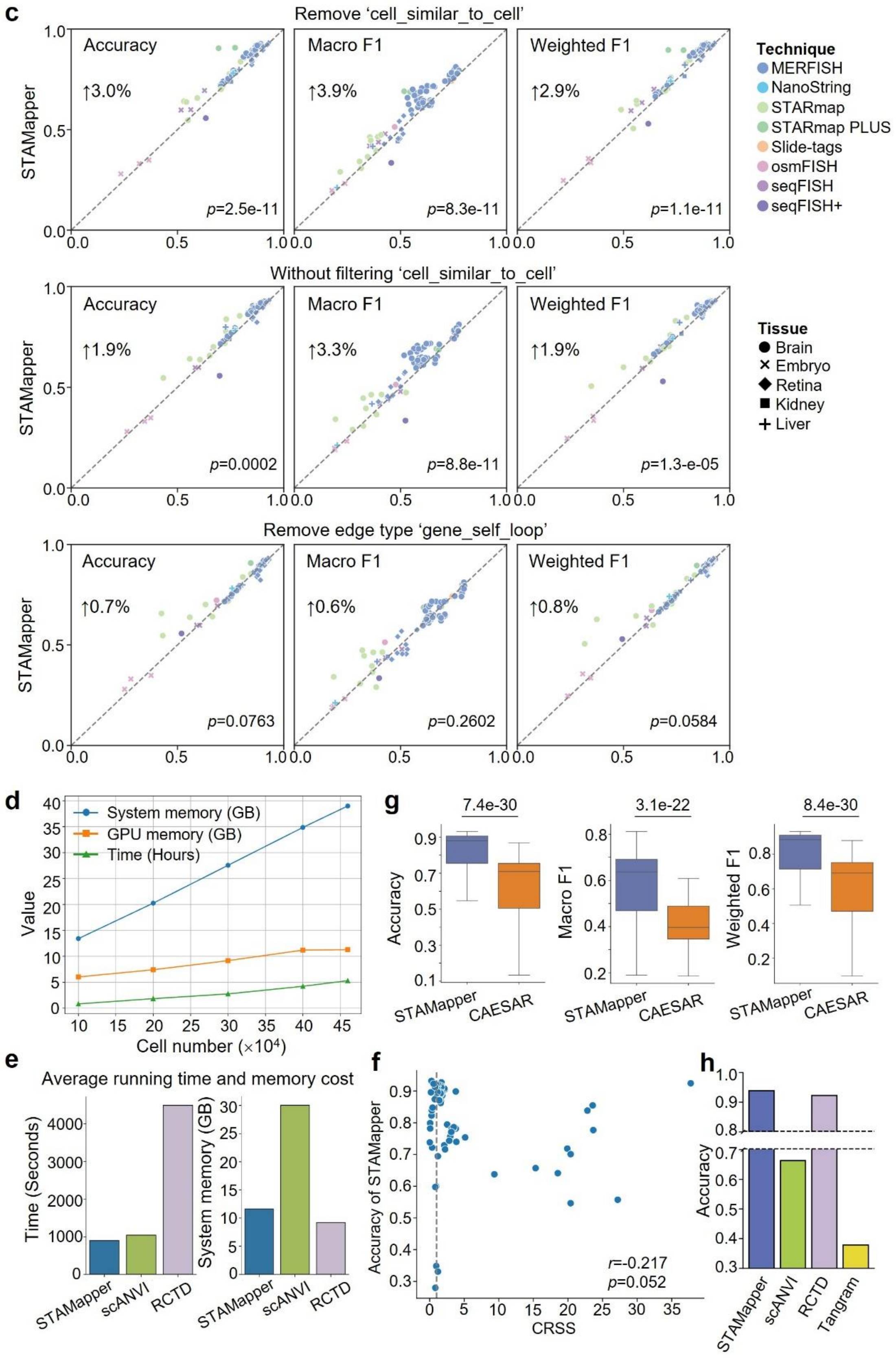

Fig. S6. a. Performance comparison of STAMapper (default parameters, vertical axis) and STAMapper with spatial information regarding cell-annotation accuracy, macro F1

score, and weighted F1 score on 81 pairs of scRNA-seq and scST datasets. For STAMapper with spatial information, we define a new edge type, "cell_near_cell", to connect spatially adjacent cells as neighbors (n=5). *P*-values were calculated using a paired t-test. **b.** Performance comparison of STAMapper (default parameters, vertical axis) and STAMapper under different settings: hidden unit sizes of 128, 256, 1024, and three hidden layers, regarding cell-annotation accuracy, macro F1 score, and weighted F1 score on 81 pairs of scRNA-seq and scST datasets. *P*-values were calculated using a paired t-test. **c**. Performance comparison of STAMapper (default parameters, vertical axis) and STAMapper under different settings: STAMapper without edge type “cell_similar_to_cell”, STAMapper without filtering edge type “cell_similar_to_cell” that connects different cell types, and STAMapper without edge type “gene_self_loop”. *P*-values were calculated using a paired t-test. **d**. Line plot illustrating how STAMapper’s runtime, peak GPU memory, and peak system RAM scale with dataset size. The NanoString human liver cancer scST dataset (460k cells) was used as the query dataset, with a scRNA-seq reference dataset of 34k cells as a benchmark. The query dataset was randomly downsampled to 100k, 200k, 300k, and 400k cells for testing runtime and peak memory usage at each scale. **e**. The average runtime and memory usage of STAMapper, scANVI, and RCTD across a total of 81 scST datasets. All analyses were run on a Linux workstation powered by an AMD EPYC 7K62 48-core CPU, an NVIDIA GeForce RTX 4090 GPU with 24 GB VRAM, and 1024 GB of system RAM. **f**. Scatterplot showing the correlation between the accuracy of STAMapper and the cell ratio between scRNA-seq reference and spatial data (CRSS), with the number of cells in the reference dataset divided by the number of cells in the spatial dataset. *P* value was calculated using Pearson correlation. **g**. Performance comparison of STAMapper and CARSAR on 80 datasets. The NanoString HCC dataset was omitted because CAESAR’s runtime on it exceeded three days, and the system memory cost was over 1024 GB. *P*-values were calculated using paired t-tests. **h**. The Accuracy of STAMapper, scANVI, RCTD, and Tangram on the COAD Xenium dataset.

Supplementary Tables

Table S1: Description of all scRNA-seq datasets used in this study.

Technique	Tissue	Description	#Genes	#Data sets	Reference
**Droplet-microfluidic**	Mouse brain	Hypothalamic preoptic region	20320	1	[2]
**10X Chromium**	Mouse kidney	Kidney	20138	1	[3]
**10x Chromium**	Mouse liver	Liver	20138	1	[3]
**Smart-seq**	Mouse brain	Visual cortex	34041	1	[4]
**10X Chromium**	Mouse brain	Prefrontal cortex	19517	1	[5]
**10X Chromium**	Mouse brain	Primary motor cortex	26523	1	[6]
**STRT/C1**	Mouse brain	Somatosensory cortex	19972	1	[7]
**10X Chromium**	Mouse retina	Retina	27998	5	[8]
**10X Chromium**	Mouse embryo	Gastrulation	19362	1	[9]
**10X Chromium**	Mouse brain	Prefrontal cortex	21000	1	[5]
**10X Chromium**	Human brain	Prefrontal cortex	26747	1	[10]
**10X Chromium**	Human liver	Hepatocellular Carcinoma	25479	1	[11]

Table S2: Description of all scST datasets used in this study.

Technique	Tissue	Description	#Genes	#Data sets	Reference
**STARmap**	Mouse brain	Prefrontal cortex	166	4	[12]
**STARmap**	Mouse brain	Visual cortex	166	3	[12]
**STARmap**	Mouse brain	Visual cortex	1020	2	[12]
**seqFISH+**	Mouse brain	Visual cortex	10000	1	[13]
**osmFISH**	Mouse brain	Somatosensory cortex	33	1	[14]
**MERFISH**	Mouse brain	Primary motor cortex	254	12	[15]
**MERFISH**	Mouse retina	Retina	368/500	10	[16]
**MERFISH**	Mouse kidney	Kidney	307	1	[3]
**MERFISH**	Mouse liver	Liver	307	1	[3]
**MERFISH**	Mouse brain	Hypothalamic preoptic region	161	36	[2]
**osmFISH**	Mouse embryo	Gastrulation	33	3	[9]
**seqFISH**	Mouse embryo	Gastrulation	251	3	[9]
**STARmap PLUS**	Mouse brain	Prefrontal cortex	2766	2	[17]
**Slide-tags**	Human brain	Prefrontal cortex	36601	1	[18]
**NanoString**	Human liver	Hepatocellular Carcinoma	1000	1	[19]

Reference

1. Liu J, Liu W, Chai X, Zhang X, Lin Z. CAESAR: a cross-technology and cross-resolution framework for spatial omics annotation. 2024.
2. Moffitt JR, Bambah-Mukku D, Eichhorn SW, Vaughn E, Shekhar K, Perez JD, et al. Molecular, spatial, and functional single-cell profiling of the hypothalamic preoptic region. Science. 2018;362(6416).
3. Liu J, Tran V, Vemuri VNP, Byrne A, Borja M, Kim YJ, et al. Concordance of MERFISH spatial transcriptomics with bulk and single-cell RNA sequencing. Life Sci Alliance. 2023;6(1).
4. Hodge RD, Bakken TE, Miller JA, Smith KA, Barkan ER, Graybuck LT, et al. Conserved cell types with divergent features in human versus mouse cortex. Nature. 2019;573(7772):61-8.
5. Bhattacherjee A, Djekidel MN, Chen R, Chen W, Tuesta LM, Zhang Y. Cell type-specific transcriptional programs in mouse prefrontal cortex during adolescence and addiction. Nat Commun. 2019;10(1):4169.
6. Yao Z, Liu H, Xie F, Fischer S, Adkins RS, Aldridge AI, et al. A transcriptomic and epigenomic cell atlas of the mouse primary motor cortex. Nature. 2021;598(7879):103-10.
7. Zeisel A, Munoz-Manchado AB, Codeluppi S, Lonnerberg P, La Manno G, Jureus A, et al. Brain structure. Cell types in the mouse cortex and hippocampus revealed by single-cell RNA-seq. Science. 2015;347(6226):1138-42.
8. Hoang T, Wang J, Boyd P, Wang F, Santiago C, Jiang L, et al. Gene regulatory networks controlling vertebrate retinal regeneration. Science. 2020;370(6519).
9. Pijuan-Sala B, Griffiths JA, Guibentif C, Hiscock TW, Jawaid W, Calero-Nieto FJ, et al. A single-cell molecular map of mouse gastrulation and early organogenesis. Nature. 2019;566(7745):490-5.
10. Herring CA, Simmons RK, Freytag S, Poppe D, Moffet JJD, Pflueger J, et al. Human prefrontal cortex gene regulatory dynamics from gestation to adulthood at single-cell resolution. Cell. 2022;185(23):4428-47 e28.
11. Lu Y, Yang A, Quan C, Pan Y, Zhang H, Li Y, et al. A single-cell atlas of the multicellular ecosystem of primary and metastatic hepatocellular carcinoma. Nat Commun. 2022;13(1):4594.
12. Wang X, Allen WE, Wright MA, Sylwestrak EL, Samusik N, Vesuna S, et al. Three-dimensional intact-tissue sequencing of single-cell transcriptional states. Science. 2018;361(6400).
13. Eng CL, Lawson M, Zhu Q, Dries R, Koulena N, Takei Y, et al. Transcriptome-scale super-resolved imaging in tissues by RNA seqFISH. Nature. 2019;568(7751):235-9.
14. Codeluppi S, Borm LE, Zeisel A, La Manno G, van Lunteren JA, Svensson CI, Linnarsson S. Spatial organization of the somatosensory cortex revealed by osmFISH. Nat Methods. 2018;15(11):932-5.
15. Zhang M, Eichhorn SW, Zingg B, Yao Z, Cotter K, Zeng H, et al. Spatially resolved cell atlas of the mouse primary motor cortex by MERFISH. Nature. 2021;598(7879):137-43.
16. Choi J, Li J, Ferdous S, Liang Q, Moffitt JR, Chen R. Spatial organization of the mouse retina at single cell resolution by MERFISH. Nat Commun. 2023;14(1):4929.
17. Zeng H, Huang J, Zhou H, Meilandt WJ, Dejanovic B, Zhou Y, et al. Integrative in situ mapping of single-cell transcriptional states and tissue histopathology in a mouse model of Alzheimer's disease. Nat Neurosci. 2023;26(3):430-46.

18. Russell AJC, Weir JA, Nadaf NM, Shabet M, Kumar V, Kambhampati S, et al. Slide-tags enables single-nucleus barcoding for multimodal spatial genomics. Nature. 2024;625(7993):101-9.
19. Hernandez S, Lazcano R, Serrano A, Powell S, Kostousov L, Mehta J, et al. Challenges and Opportunities for Immunoprofiling Using a Spatial High-Plex Technology: The NanoString GeoMx((R)) Digital Spatial Profiler. Front Oncol. 2022;12:890410.
